# The Role of Cyclic Diketopiperazine in the Formation of Polypeptides on Silica Surfaces

**DOI:** 10.1002/cplu.70216

**Published:** 2026-07-26

**Authors:** Ola El Samrout, Rita Arnesi, Samuele Mistrali, Chiara Nannuzzi, Gloria Berlier

**Affiliations:** ^1^ Department of Chemistry NIS and INSTM Centers University of Turin Torino Italy

**Keywords:** cyclic dimer, glycine, linear peptides, polymerization, silica

## Abstract

Diketopiperazine (DKP), a recurring product of silica‐catalyzed amino acid polymerization, is a molecule of interest in the origin of life studies, as well in the industrial medicinal chemistry. Its role in the peptide bond formation reactions is controversial as it is considered sometimes as a stable, dead‐end product for the oligomerization reaction. We tried to elucidate the parameters that govern the DKP opening on silica surfaces of low and high surface areas in the frame of glycine polymerization reaction, when DKP is adsorbed from gas and liquid phases, or subjected to temperature and humidity fluctuations cycles. The formed products were characterized by infrared spectroscopy, thermogravimetric analysis, and X‐ray diffraction. The results reveal that DKP represents an efficient intermediate for the polymerization reaction leading to the formation of linear peptides on silica surfaces. Abundant oligomers with β‐sheet secondary structures are formed depending on the DKP loading and the silica surface. The crucial role of the silica surface including the “nearly free” silanols in the adsorption, opening, and reaction of DKP to form linear chains was shown. These conclusions highlight the complexity of the DKP surface chemistry in the polymerization reaction and can improve the understanding when dealing with geochemical prebiotic scenarios.

## Graphical abstract

The parameters governing diketopiperazine (DKP) deposition, reactivity, and opening on amorphous silica are investigated through complementary studies in both liquid and gas phases in the frame of glycine (Gly) polymerization. The results demonstrate that DKP acts as an efficient intermediate toward the formation of linear poly‐Gly, yielding significantly higher amounts of polymers compared with silica surface where DKP is not involved. For the gas phase deposition, the interaction between DKP and nearly free silanols (NFS) plays a crucial role in the adsorption and reactivity of DKP on the silica surface.

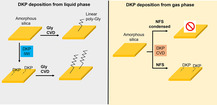

## 1 Introduction

Cyclic dipeptides, also known as diketopiperazines (DKPs), are a class of organic compounds originating from the cyclization of a dipeptide into six‐membered heterocycle with two amide bonds. They are considered as the simplest, naturally occurring cyclic forms of peptides and can be found in many living beings—from bacteria to mammals—as biosynthesis products [1]. DKPs exist in the form of three possible regio‐isomers that differ from the relative position of the two carbonyl groups: 2,3‐, 2,5‐ and 2,6‐DKPs [2, 3, 4]. Among these isomers, 2,5‐DKP, simply known as DKP or glycine anhydride (the cyclic form of the glycine dimer), has attracted the most attention, owing to its peculiar heterocyclic system, found in several natural products, which constitutes a rich source of new biologically active compounds [1]. DKP is characterized by a conformational rigidity and a proteolysis‐resistant structure that makes it an optimal compound for combinatorial chemistry [5]. Moreover, its hydrogen‐bonding capabilities favor interaction with a large variety of biological targets: In fact, they are known to exhibit antitumoral, antiviral, and antibacterial activities. For all these reasons, DKP has been the subject of pharmacological research in the last years, leading to the discovery of promising agents for drug development [3, 6, 7, 8].

In addition to its crucial role in pharmacology and industrial medicinal chemistry, DKP has also attracted a high interest in prebiotic chemistry. DKPs can be formed abiotically from basic amino acids under prebiotic conditions, which implies that they may have been among the earliest peptides to emerge on the primitive Earth, providing valuable clues about the transition from simple organic compounds to more complex biomolecules [9]. DKPs are easily formed, sometimes as a major product, in most experiments concerning prebiotic peptide formation. Basiuk and coworkers studied polymerization from gas phase of several proteogenic amino acids, including glycine (Gly), on thermally pretreated silica supports of ∼300 m^2^.g^−1^. In earlier experiments, amino acid deposition was carried out by sublimation at ∼250 °C under vacuum and was found to yield DKP as a major product [10]. In subsequent works, the sublimation procedure was refined: In the case of Gly, sublimation temperatures were lowered to 170–180 °C, and reagent quantities were increased from milligrams to grams. DKP was again observed as the main product [11]. Bujdàk and Rode [12, 13] applied drying and wetting cycles to Gly on silica supports of unknown surface area. Amino acids were deposited from a reactant solution; the system was then dried and thermally activated at 80–85 °C. Overall, both linear and cyclic dimers were observed as products of concurrent reactions, but DKP was found to be marginally predominant at higher reaction times and low‐water conditions. It should be noted, however, that yields were particularly low (under 0.9%), presumably due to the low reaction temperatures.

Despite being a predominant product in most of the peptide bond formation reactions, the role of DKP in the polymerization reaction of amino acids represents a subject of debate with differing outcomes. Some research groups believe that DKP is a stable, undesirable side product of amino acid polymerization, incapable of promoting successive chain growth due to its resistance to proteolysis; while other groups, on the other hand, suggest that DKP is, in fact, an intermediate for peptide formation.

Experiments conducted by Lambert et al. [14] on high surface area silica supports, specifically A380 (380 m^2^.g^−1^), showed significant DKP formation between 150–200 °C, confirming that higher temperatures are required to sustain the dimerization process. Yet, contrary to Bujdàk and Rode's findings [12], no trace of linear dimers was detected: This discrepancy was explained by considering Gly–Gly chains as products of DKPs' hydrolytic opening caused by the re‐introduction of water from the hydration step. Later, experiments conducted in similar conditions [15, 16] confirmed the predominance of DKP as a product when the reaction is carried out under dry air. Such results led the authors to the idea that DKP could constitute an intermediate for linear oligomer synthesis, rather than a dead‐end product. Nagayama et al. [17] researched this matter and determined that DKP opening and subsequent oligomerization can indeed occur in aqueous solutions of DKP and Gly monomers, dimers, or trimers after thermal activation at 90 °C. Under these conditions, the observed products were Gly trimers, tetramers, and pentamers respectively: This is explained by the amino acid moiety of a Gly molecule or oligomer performing a nucleophilic attack on the DKP, causing it to open. For this reason, the resulting peptide is two Gly units longer than its noncyclic reagent. It should be noted that silica is not involved in this study; hence, no catalytic effect is at play.

It was not until some years later that this topic was further inquired by Lambert's group [15], who noted that conditions of fluctuating humidity (i.e., wetting and drying (W/D) cycles), as seen in Bujdák and Rode's studies [12], on Gly/silica systems favor DKP opening by hydrolysis, leading to the formation of linear peptides in addition to cyclic dimers. Their experiment consisted of seven iterations of a drying step at either 85 or 135 °C followed by a wetting step with liquid water on Gly/silica samples. While no conclusions could be inferred from the results at 85 °C due to low amounts of product, which were attributed to slow reaction rates, activation at 135 °C resulted in stable quantities of DKP after the first cycle and increasing amounts of GlyGly after each cycle, corroborating the authors’ hypothesis. In addition, longer peptides were also detected, the most abundant being tetraGly, which is thought to be a product of condensation between adsorbed GlyGly and DKP.

**SCHEME 1:**
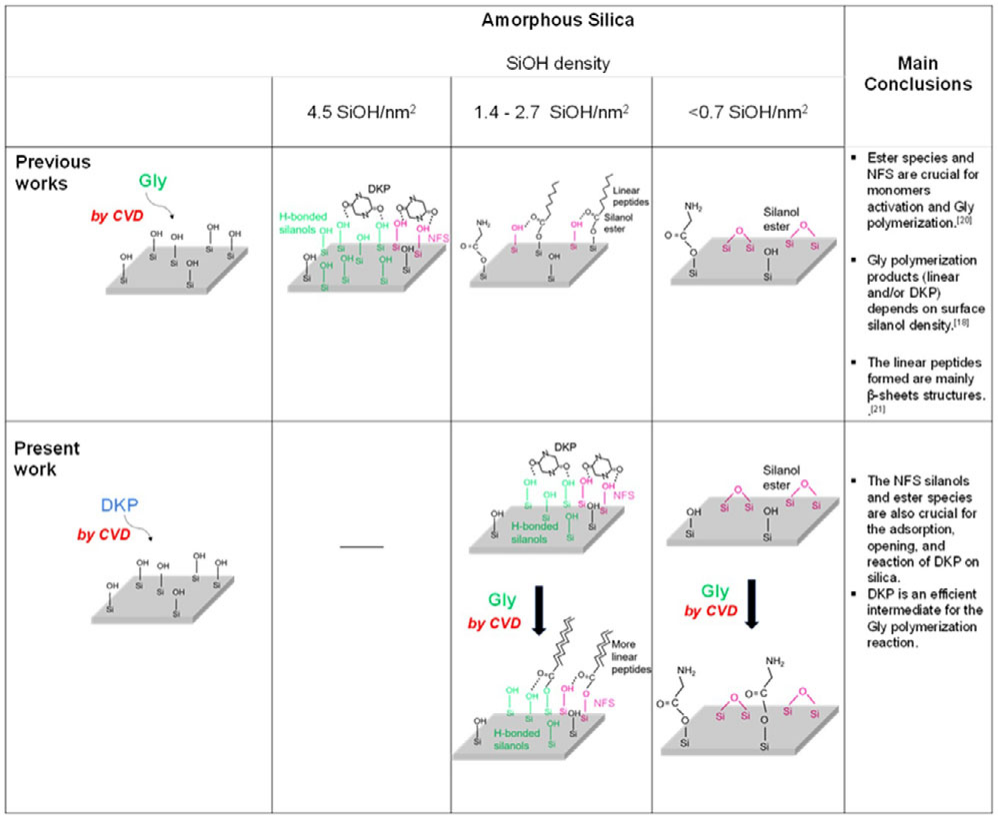
Schematic representation of the main findings obtained in our earlier studies [18, 20, 21] and the present one.

In subsequent works, some of us [18] aimed to shed light on the effects of silanol density, deposition technique, and amino acid loading on the product of Gly polymerization reaction (linear peptides or cyclic DKP). Experiments were carried out on silica samples of varying surface areas on which Gly deposition was achieved by adsorption either from vapor using chemical vapor deposition (CVD) or aqueous phases using incipient wetness impregnation (IWI). It was concluded that DKP formation is favored on A380 silica while linear peptides are formed on A50 (50 m^2^.g^−1^) silica when amino acids are deposited from vapor phase (Scheme 1). The rationale behind this behavior can be explained considering the role of a special type of silanol groups distant by 4–6 Å called nearly free silanols or NFSs [19] as both primers and aiding elements for chain elongation. This study highlights that silanol density is correlated to surface area, so the optimum number of NFSs on a given silica support is achieved if the surface area falls under a precise range of values. This way, silanols are neither too dispersed (isolated) nor too close (hydrogen‐bonded) but loosely interact with each other (nearly free). However, the mechanism and crucial parameters for DKP opening on silica surface in the frame of the polymerization reaction are still poorly understood.

Based on the limited number of scientific papers in literature to study the experimental conditions for DKP opening on silica surface for the peptide bond formation, the aim of the present work is to study different experimental attempts for DKP opening on both low‐ and high‐surface area silica surfaces in the frame of the Gly polymerization reaction where DKP is deposited either (1) from liquid phase using IWI, (2) from gas phase using CVD, or (3) subjected to conditions of temperature and humidity fluctuations (W/D cycles). In addition, the work focuses on the role of silanol groups on silica in DKP opening as well as the secondary structures and mobility of the resulting polymerization reaction product. The study builds upon our earlier investigations that (i) identified the cooperative role of ester species and NFSs as essential surface motifs for amino acid activation and peptide formation on silica [20], (ii) demonstrated that dynamic humidity fluctuations promote peptide growth and structural reorganization into stable β‐sheet‐rich assemblies [21], and (iii) identified the silica‐surface parameters controlling whether Gly polymerization yields linear peptides or DKP [18]. These findings are summarized in Scheme 1, which highlights the differences between our earlier studies and the present work.

## 2 Results and Discussion

Two types of fumed silica surfaces of low and high surface area (Aerosil A50 and A380, respectively) were used to study the effect of silica surface and experimental conditions on the opening of the cyclic anhydride DKP. These silicas have been used by some of us in a previous work as platform for the polymerization of Gly after deposition from liquid or gas phases [18]. DKP was deposited either from liquid phase using IWI and different weight loadings, or from gas phase through CVD. Using IWI procedure allows to impose the weight loading of DKP deposited on the silica surface. This is not possible by CVD; however, this approach allows us to minimize the influence of water and to study the role of surface silanols in the process [18, 20, 21, 22, 23].

After DKP deposition, different attempts were made for its opening including prolonged thermal activation, wetting/drying cycles, postdeposition of Gly monomers by CVD or IWI, or deposition on a surface after initial reaction of this surface with Gly. The secondary structures and structural dynamics of the resulting Gly oligomers, when formed, were studied, and the role of silica surface sites in opening of DKP have been investigated.

### 2.1 DKP Deposited on Silica From Liquid Phase by IWI

#### 2.1.1 Thermal Activation

First, we have considered the effect of thermal activation on the ring opening of DKP adsorbed on the silica surface. For this aim, a sample, labeled as DKP_2(IWI)_/A50, has been prepared by the deposition of 2 wt% of DKP on silica A50 by IWI as explained in Section 4, followed by an outgas under vacuum at 140 °C for 30 min to remove the part of DKP units that precipitated in the form of bulk crystals. This DKP loading corresponds roughly to a monolayer of DKP. The calculation is based on an estimated area of DKP molecule of 51.66 Å^2^ (when considering that DKP is occupying a rectangle shape with a length of 8.6 Å and a width of 5–6 Å) [24], “lying flat” on the surface of silica in a closed‐packed arrangement. This corresponds to roughly 1.9 DKP⋅nm^−2^, which accounts for ca. 1.6 wt% on A50.

After detecting the adsorption of DKP on A50 by IWI using infrared (IR) spectroscopy, the sample was subjected to thermal activation at 160 °C for 2.5 h under vacuum. The corresponding IR profiles measured after each step are displayed in Figure S1. The IR profile recorded after IWI and outgas (Figure S1, curve a) shows the characteristic bands of DKP including the amide I and DKP ring‐stretching bands (1674 and 1468 cm^−1^, respectively) and the amide A band at 3390 cm^−1^ (see Table 1 for the assignment the main IR bands observed in this work). This indicates the adsorption of DKP on the surface of silica A50 [18]. However, after thermal activation at 160 °C for 2.5 h under vacuum, we observe the disappearance of the bands related to DKP (Figure S1, curve b) indicating its desorption from the surface. In the *ν*_OH_ region, the silanol pattern is affected by the desorption of DKP molecules from the surface: A recovery of the silanols profile peaking at 3744 cm^−1^ where weakly interacting silanols, known as NFS, are found [20, 25]. This suggests that NFS groups are involved in the adsorption of DKP on the silica surface. Their role in DKP adsorption and ring opening will be discussed in detail in the following section, when addressing the deposition of DKP by CVD on the same silica sample.

**TABLE 1:** List of bands assignments for IR spectra.

Wavenumber, cm^−1^	IR vibration
3747	*ν*_OH_ of isolated silanol groups
3744–3743	*ν*_OH_ of NFS groups
3400–3280	*ν*_NH_ Amide A of DKP ring
3315–3302	*ν*_NH_ Amide A of peptide chains
3185	*ν*_NH_ of Gly monomers
3085–3080	*ν*_NH_ Amide B of peptide chains
1750–1745	*ν*_co_ of ester groups
1680–1670	*ν*_co_ Amide I of adsorbed DKP
1670–1655	*ν*_co_/*δ*_NH_ Amide I of peptide chains
1580–1470	*δ*_NH_/*ν*_CN_ Amide II of peptide chains
1472–1468	DKP ring stretching
1463	Amide II’ of deuterated peptides

#### 2.1.2 Wetting/Drying Cycles

To study the effect of humidity fluctuations on the opening of DKP ring on silica surface, wetting/drying cycles were applied on silica A380 sample after adsorption of 4% DKP by IWI. Based on the calculations described above, this loading corresponds to a coverage of 0.33, the monolayer on A380 accounting to 12.16 wt%. The prepared sample labeled as DKP_4(IWI)_/A380 was subjected to water vapor admission for 20 min followed by a heating under vacuum at 80 °C for 30 min while in contact with water vapor. Subsequently, the sample was dried under vacuum at rt and then heated at 80 °C for 30 min. The cycles of wetting/drying (W/D) are repeated until invariance of spectra. The IR spectra recorded before and at the end of the W/D cycles are displayed in Figure S2A.

The IR profiles of both DKP_4(IWI)_/A380 recorded after DKP deposition (curve a) and at the end of the W/D cycles applied (curve b) show no evident change in the characteristic bands of DKP adsorbed on the surface: the amide I (1679–1675 cm^−1^) and the DKP ring stretching (1470 cm^−1^) remain almost unaltered at the end of the W/D cycles. The persistence of these bands along with the absence of the formation of amide II band indicate the resistance of the DKP ring to opening by hydrolysis, and consequently the absence of the oligomers formation by W/D cycles [18].

As further confirmation of what is present on the surface after the W/D cycles, the sample was subjected to TGA measurements. The corresponding derivative thermogravimetric (DTG) trace is reported in Figure S3A. A detailed explanation about the information that can be obtained by this technique in this context is reported in the following section. In this section, it is only important to acknowledge the fact that one single thermal event is observed at around 260 °C which is associated with the elimination of molecularly adsorbed DKP molecules [14]. For comparison, the same experiment was carried out after deposition of DKP by CVD, resulting in sample DKP_(CVD)_/A380 (part B in Figures S2 and S3). The results are similar, apart from a difference in the final DKP loading calculated from the weight loss (28.24 vs. 2.96 for the sample prepared by IWI) and the presence of a minor thermal event at 332 °C related to the degradation of crystalline bulk DKP (Table S1).

The resistance of DKP rings to hydrolysis in this section may seem in contradiction with previous studies carried by Bujdàk and Rode [26] and Georgelin et al. [15]; however, it should be noted that the aforementioned works performed the W/D cycles with liquid water (using around 10 ml) and/or by heating the system at higher temperature (135 °C) for 1 day long. In such conditions, it is rational to assume that DKP hydrolysis rate was augmented, thus leading to the formation of linear Gly–Gly.

#### 2.1.3 Reaction With Gly Monomers Dosed From the Gas Phase

Two sets of samples labeled as DKP_*x*(IWI)_/A50 and DKP_*x*(IWI)_/A380 were prepared using the two different silica substrates A50 and A380 where various DKP weight loadings (designated as *x* = 1, 2, and 3 wt%) were deposited using IWI. An additional sample with 4 wt% DKP loading was prepared using A380 as substrate.

**FIGURE 1:**
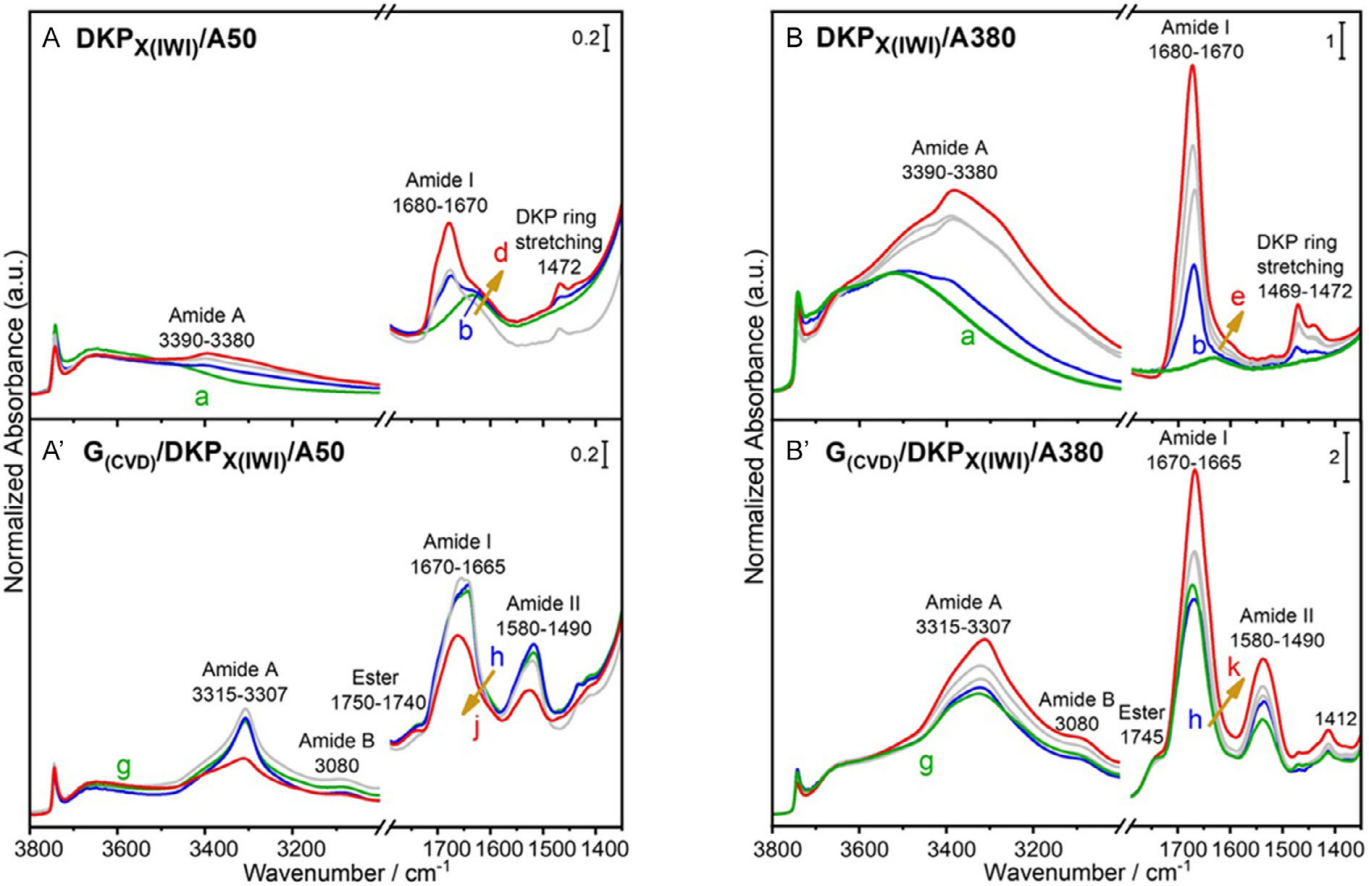
IR spectra measured on self‐supporting pellets resulting from DKP deposition by IWI on silica surfaces: (A) DKP_*x*(IWI)_/A50 followed by an outgas at 140 °C for 30 min and (B) DKP_*x*(IWI)_/A380 followed by an outgas at room temperature for 2 h. Subsequently, the IR spectra measured after Gly deposition by CVD under vacuum for 2.5 h on each of the different pellets are represented in panels: (A’) G_(CVD)_/DKP_*x*(IWI)_/A50 and (B’) G_(CVD)_/DKP_*x*(IWI)_/A380; where *x* in all panels refers to different DKP loadings ranging from (b) or (h) 1 wt% to (d) or (j) 3 wt% for silica A50, and from (b) or (h) 1 wt% to (e) or (k) 4 wt% for silica A380. The IR spectra (a) and (g) refer to the corresponding bare silica A50 and A380 pellets for the sake of comparison obtained from distilled water by IWI and outgas (Panels A and B), then the subsequent 2.5 h Gly sublimation (Panels A’ and B’), respectively.

**FIGURE 2:**
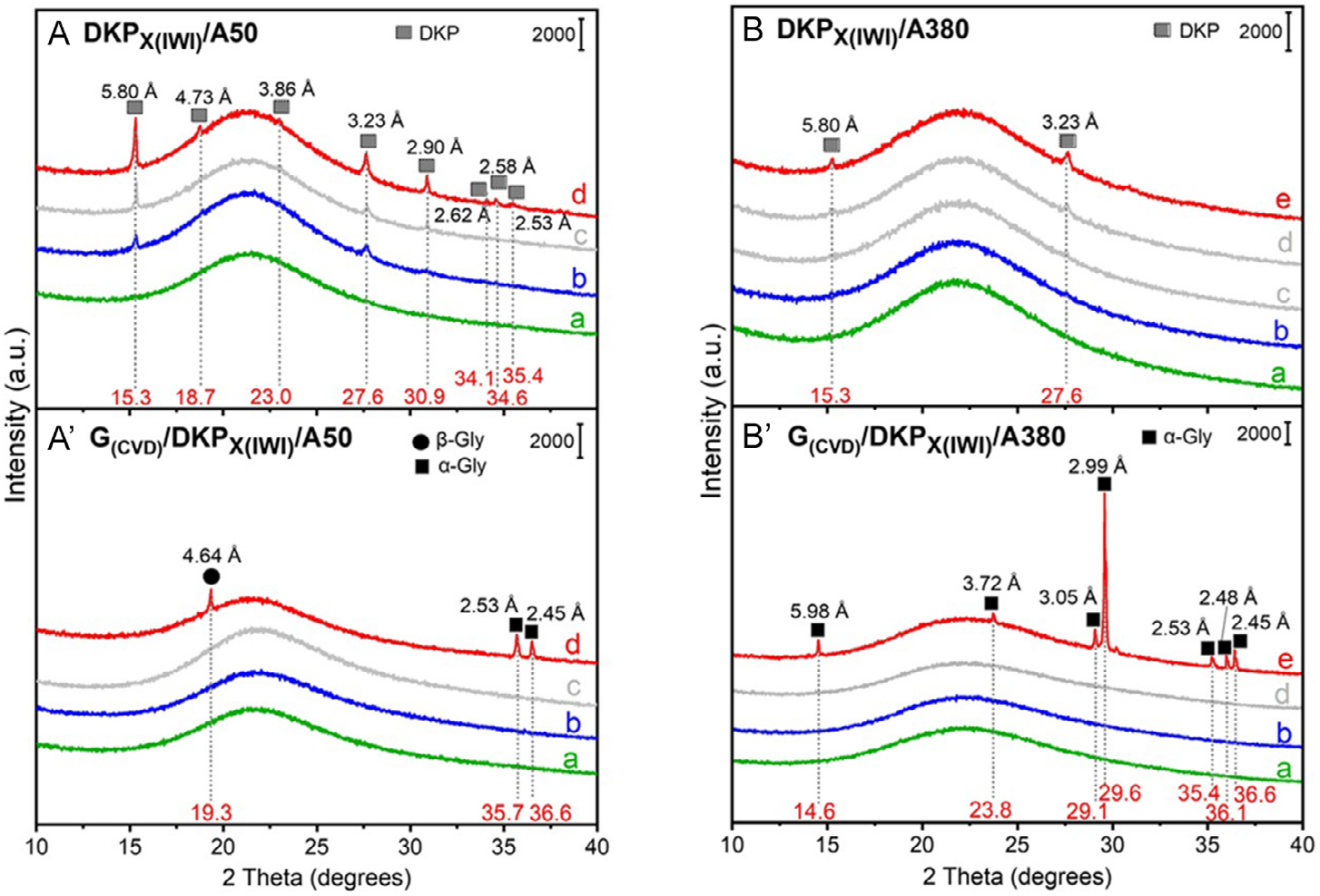
XRD profiles measured on self‐supporting pellets resulting from DKP deposition by IWI on silica surfaces: (A) DKP_*x*(IWI)_/A50 followed by an outgas at 140 °C for 30 min and (B) DKP_*x*(IWI)_/A380 followed by an outgas at room temperature for 2 h. Subsequently, the XRD profiles measured after Gly deposition by CVD under vacuum for 2.5 h on each of the different pellets are represented in panels: (A’) G_(CVD)_/DKP_*x*(IWI)_/A50 and (B’) G_(CVD)_/DKP_*x*(IWI)_/A380; where *x* in all panels refers to different DKP loadings: (b) 1, (c) 2, (d) 3, and (e) 4 wt%. The XRD profiles (a) refer to the corresponding bare silica A50 and A380 pellets for the sake of comparison obtained from: distilled water impregnation by IWI and outgas (Panels A and B), then the subsequent 2.5 h Gly sublimation (Panels A’ and B’), respectively.

**FIGURE 3:**
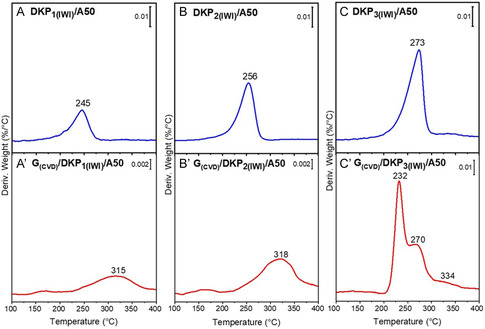
Derivative thermograms (DTG) for samples obtained after DKP deposition by IWI and outgas on silica A50 surfaces: (A) DKP_1(IWI)_/A50, (B) DKP_2(IWI)_/A50, (C) DKP_3(IWI)_/A50; and subsequently after Gly sublimation by CVD for 2.5 h under vacuum: (A’) G_(CVD)_/DKP_1(IWI)_/A50, (B’) G_(CVD)_/DKP_2(IWI)_/A50, (C’) G_(CVD)_/DKP_3(IWI)_/A50.

After IWI deposition, all samples were outgassed at room temperature for 2 h. In addition, only the samples with silica A50 were further outgassed at 140 °C for 30 min to remove the part of DKP that precipitated in the form of bulk crystals. Subsequently, Gly monomers were adsorbed from gas phase using CVD on each of these samples as an attempt for DKP opening on silica. Gly has been selected as this is the simplest amino acid, and it represents a reference molecule for polymerization studies on silica without the complexity introduced by the lateral substituent [18, 20, 21, 22]. The new obtained sets were labeled as G_(CVD)_/DKP_*x*(IWI)_/A50 and G_(CVD)_/DKP_*x*(IWI)_/A380. The IR spectra, XRD profiles, and DTG thermograms of the different sets of samples are presented in Figures 1, 2, and 3 respectively.

The IR spectra of the A50 and A380 silicas, measured after distilled water impregnation by IWI and outgas, are shown in Figure 1A,B curve a, respectively. The two silicas show a different intensity and distribution of Si‐OH groups, as seen in the high wavenumbers region, because of the different surface areas. At low wavenumbers, a broad peak is seen at 1633 cm^−1^, related to a combination mode of SiO_4_ vibrations.

After DKP deposition by IWI on silica surfaces A50 and A380 followed by outgas, the resulting IR spectra of DKP_*x*(IWI)_/A50 and DKP_*x*(IWI)_/A380 sets (Figure 1A, curves b–d and Figure 1B, curves b–e) show the formation at low wavenumbers of a band in the 1680–1670 cm^−1^ range attributed to *ν*_CO_ of adsorbed DKP, known as amide I; along with a band around 1472 cm^−1^ associated to DKP ring stretching (Table 1) [18, 27, 28]. At high wavenumbers, the IR profiles of both sets of samples display a band in the 3390–3380 cm^−1^ range which corresponds to *ν*_NH_ of the DKP ring, designated as amide A [14]. The aforementioned bands show a gradual increase in intensity with the increase of DKP weight loadings (from 1 to 3 wt% in Figure 1A and from 1 to 4 wt% in Figure 1B) on both silica substrates A50 and A380, as expected.

Each sample in both sets was then subjected to Gly monomers adsorption by CVD at 160 °C for 2.5 h. Figure 1A’,B’ shows the IR spectra recorded after 2.5 h CVD for the samples obtained, labeled as G_(CVD)_/DKP_*x*(IWI)_/A50 (Figure 1A’) and G_(CVD)_/DKP_*x*(IWI)_/A380 (Figure 1B’). At low frequency, the amide I shifted to lower wavenumber (1670–1665 cm^−1^), and amide II (1580–1490 cm^−1^) bands are observed for both sets of samples using A50 and A380 as substrates (curves h–j in Figure 1A’ and curves h–k in Figure 1B’, respectively). The formation of the amide I and amide II bands along with the absence of the characteristic bands of DKP on the IR profiles recorded after 2.5 h Gly CVD (Figure 1A’,B’) is an indication of the opening of DKP ring into linear peptide chains rather than being simply desorbed as TG data (Figure 3) indicate that DKP desorbs/degrades only at a 100 °C higher temperature. In addition, the appearance of the band in the range of 1750–1740 cm^−1^ indicates the formation of ester groups between the linear peptides and surface silanols [18, 20, 21, 23]. Further confirmations of the opening of DKP rings into linear peptide chains can be found, at high frequency, in the shift at lower wavenumber and narrowing in shape of amide A (3315–3307 cm^−1^) along with the formation of amide B (3080 cm^−1^) bands that arise from the ν_NH_ in the peptide chains [20, 21].

For G_(CVD)_/DKP_*x*(IWI)_/A50 series (Figure 1A’), the intensities of amide I and amide II bands decrease with the increase of the DKP weight loading predeposited on the sample prior to Gly CVD (from 0 wt% for the A50 reference sample, curve g to 3 wt% for the G_(CVD)_/DKP_3(IWI)_/A50 sample, curve j Figure 1A’). The intensity of amide A lowers, and its shape becomes broader with high DKP weight loading (3 wt% DKP, curve j in Figure 1A’).

On the contrary, for G_(CVD)_/DKP_*x*(IWI)_/A380 series (Figure 1B’), a different scenario is observed: The intensities of amide I and amide II bands progressively increase with the increase of DKP weight loading predeposited by IWI (from 0 wt% for the A380 reference sample, curve g to 4 wt% for the G_(CVD)_/DKP_4(IWI)_/A380, curve k). This is also coherent with the behavior of amide A on A380 substrate: Amide A band increases in intensity and narrows in shape as more DKP weight loading is predeposited on the surface by IWI.

The relative amount of peptides formed after DKP opening may be evaluated from the integrated area of the amide I band (Figure S4). For both sets of samples (G_(CVD)_/DKP_*x*(IWI)_/A50 and G_(CVD)_/DKP_*x*(IWI)_/A380), the evolution of peptide band (amide I) can be roughly fitted with a straight line with nonzero intercepts. Figure S4 shows that the amount of peptides formed significantly increase with the increase (from 0–4 wt%) of DKP weight loading predeposited by IWI before the 2.5 h Gly CVD on the A380 substrate; while the amount of peptide chains is lower when higher DKP weight loading is prior deposited to CVD on the A50 substrate. Thus, the DKP predeposited by IWI on A380 of high surface area (380 m^2^.g^−1^) acts as an efficient intermediate product that promotes the significant formation and growth of linear peptides on silica, while on A50 of low surface‐area (50 m^2^.g^−1^), and increasing the amount of DKP predeposited on the surface does not show the same benefit on the polymerization reaction. This may be related to the dearth of silanol groups to interact effectively with DKP predeposited for its opening by Gly CVD. This will be further discussed in the following section.

After outgas at 140 °C for 30 min (for the A50 series) or at room temperature (for the A380 series), the two sets obtained (DKP_*x*(IWI)_/A50 and DKP_*x*(IWI)_/A380) were subjected to XRD measurements, and the corresponding patterns were presented in Figure 2A,B, respectively. It is expected that DKP units are able to molecularly adsorb on the silica surface up to the saturation coverage. Above a specific loading, any additional DKP units forced to deposit will precipitate as bulk DKP.

The XRD patterns of DKP_*x*(IWI)_/A50 (Figure 2A) show peaks associated to bulk DKP (2*θ* equals 15.3°, 18.7°, 23.0°, 27.6°, 30.9°, 34.1°, 34.6°, and 35.4°, according to JCPDS file 36‐1684) starting from a low loading of 1–3 wt% (patterns b–d, respectively) compared to zero peaks for the reference A50 subjected only to outgas (pattern a). The peaks observed grow with DKP loading. For DKP_*x*(IWI)_/A380 (Figure 2B), the XRD peaks associated with crystalline DKP (2*θ* equals 15.3° and 27.6°) are only observed for the high DKP weight loadings (3 and 4 wt%, patterns d and e). This implies that DKP is molecularly dispersed only on A380 at 1 and 2 wt%, while crystalline phases are formed in all the other cases even at surface coverage below the saturation coverage.

After 2.5 h Gly CVD, no XRD peaks associated with crystalline DKP are further observed on any of the samples (Figure 2A’,B’). XRD peaks associated with bulk Gly are observed only on the samples prepared with the high DKP weight loadings (3 wt% on A50 substrate, pattern d, Figure 2A’; and 4 wt% on A380 substrate, pattern e, Figure 2B’). For G_(CVD)_/DKP_3(IWI)_/A50 (Figure 2A’), the XRD peaks observed at 2*θ* equals 19.3° may be associated to bulk β‐Gly (JCPDS file 32‐1702) whereas the ones at 35.7° and 36.6° may be assigned to α‐Gly (JCPDS file 02‐0171). The coexistence of these two phases of crystalline Gly on samples with A50 substrate has been reported before [18]; also, for A380 [14]. For G_(CVD)_/DKP_4(IWI)_/A380 (Figure 2B’), the XRD peaks at 2*θ* equals 14.6°, 23.8°, 29.1°, 29.6°, 35.4°, 36.1°, and 36.6° refer all to α‐Gly. The observation of bulk monomeric Gly species on the samples prepared with high DKP weight loadings is coherent with the fact that bulk DKP does not react efficiently to open and polymerize at 160 °C. This has been reported before for bulk Gly monomers [16] and will be discussed further for DKP in the following section.

Thermogravimetric analysis (TGA) has been used in several previous studies to identify the nature and amount of organic matter on silica supports [29, 30, 31] and particularly in the frame of the polymerization reaction studies [14, 18]. In the present study, TGA represents an accurate technique to detect the presence of DKP on the silica surface after its deposition by IWI and then its transformation or not into linear oligomers. TGA allows also to evaluate the amount of DKP adsorbed on the surface as well as that of the peptides formed afterwards. The DTG patterns of DKP_*x*(IWI)_/A50 measured after DKP deposition by IWI and outgas and of G_(CVD)_/DKP_*x*(IWI)_/A50 series obtained after 2.5 h Gly CVD at 160 °C are displayed in Figure 3. The amount of adsorbed organic matter obtained by TGA for the samples prepared with the A50 substrate are listed in Table 2, expressed weight loss % and in terms of Gly or DKP monomers.

**TABLE 2:** Table presenting the adsorbed amount of organic matter (in terms of DKP or Gly) on all the samples DKP_*x*(IWI)_/A50 obtained after DKP deposition by IWI and outgas; and subsequently on the samples G_(CVD)_/DKP_*x*(IWI)_/A50 after 2.5 h Gly sublimation by CVD. These values are calculated from the integration of DTG peaks of Figure 3 for the corresponding samples.

Sample	Peak temperature, °C	Adsorbed organic matter		
		Weight loss %	mmol DKP/g SiO_2_	mmol Gly/g SiO_2_
DKP_1(IWI)_/A50	245	0.42	0.04	—
DKP_2(IWI)_/A50	256	1.12	0.10	—
DKP_3(IWI)_/A50	273	1.94	0.17	—
G_(CVD)_/DKP_1(IWI)_/A50	315	0.53	—	0.07
G_(CVD)_/DKP_2(IWI)_/A50	318	0.63	—	0.08
G_(CVD)_/DKP_3(IWI)_/A50	232	1.86	—	0.25
	270	1.05	0.09	—
	334	0.35	—	0.05

The derivative thermograms for all the samples in Figure 3 are displayed from 100 to 400 °C. The thermal event below 100 °C corresponds to the desorption of physisorbed water from the surface, while by heating above 400 °C, all organic matter is eliminated as proved by elemental analysis. For DKP_*x*(IWI)_/A50 samples (Figure 3A–C), a single thermal event is observed in the range of 245–273 °C and which corresponds to the thermal degradation of DKP deposited on the silica surface by IWI [14, 18]. The corresponding thermal event shifts in temperature to a higher value (from 245 to 273 °C) and increases in intensity as more DKP is predeposited (from 1 to 3 wt%) on the surface. The integration of this band for each of these samples prepared with 1, 2, and 3 wt% DKP (Table 2) reveals that the actual DKP amounts present on the surface are about 0.42%, 1.12%, and 1.94% by weight of silica, respectively. This means that a part of the deposited DKP is lost on sublimation during the outgas at 140 °C for 30 min after IWI. Thus, even if the IWI deposition procedure allows us to impose the DKP loading deposited, the DKP amount present on the surface may decrease after thermal activation. This was also seen in previous studies [16, 18].

After Gly CVD for 2.5 h at 160 °C under vacuum as an attempt to open DKP deposited on the surface, the DTGs of G_(CVD)_/DKP_1(IWI)_/A50 and G_(CVD)_/DKP_2(IWI)_/A50 (Figure 3A’,B’, respectively) present only one single thermal event in the range of 315–318 °C. Such event corresponds to the oxidative degradation of glycine oligomers [16, 18, 32], and which constitute around 0.53% and 0.63% by weight of silica for G_(CVD)_/DKP_1(IWI)_/A50 and G_(CVD)_/DKP_2(IWI)_/A50, respectively (Table 2). This agrees with the IR results (Figure 1A’) where both samples showed significant formation of linear oligomers.

For G_(CVD)_/DKP_3(IWI)_/A50 (Figure 3C’), the DTG shows a different behavior as three different peaks were observed. The assignments of these three events can be deduced from combining the results of IR (Figure 1A’, curve j) and XRD (Figure 2A’, curve d) along with its DTG trace. The intense event at around 232 °C (which represents around 1.86% by weight of silica according to Table 2) could be associated with the desorption of a significant part of the Gly monomers. While the event at 270 °C could be associated with the decomposition of some amount of DKP on the surface (estimated amount 1.05% by weight of silica).

The less evident thermal event at 334 °C can be assigned to the oxidative degradation of a small fraction (only 0.35% by weight) of oligomers strongly bonded to the silica surface, in agreement with the IR results (Figure 1A’) [14, 18].

As for the G_(CVD)_/DKP_x(IWI)_/A380 samples, the TGA has been focused on the sample with high DKP loading (4 wt%), which showed the highest formation of linear peptides. This is discussed in the next section, in comparison with the results obtained by deposition of DKP by gas phase with CVD.

### 2.2 DKP Deposited on Silica From Gas Phase by CVD

#### 2.2.1 Reaction With Gly Monomers Dosed From the Gas Phase

For this set of experiments, two samples were prepared where DKP was adsorbed by CVD on silica A50 surfaces pretreated at different temperatures (160 or 700 °C) followed by Gly monomers adsorption by CVD. The samples were labeled as G_(CVD)_/DKP_(CVD)_/A50_160_ and G_(CVD)_/DKP_(CVD)_/A50_700_, respectively. The silica A50 has been selected in this section because according to our previous studies where A50 surface has been deeply studied [18, 20, 21], it constitutes an efficient platform for the formation of linear oligomers as it contains a significant number of reactive sites for the peptide formation reaction. Treating the A50 surface at different temperatures before DKP deposition allows to modulate the population of silanol and siloxane rings and study the effect of such change on the adsorption and reaction of DKP. On the other hand, CVD allows to study the gas (DKP or Gly vapor)/solid (silica) interface minimizing the influence of water [22] in contrary to IWI procedure.

**FIGURE 4:**
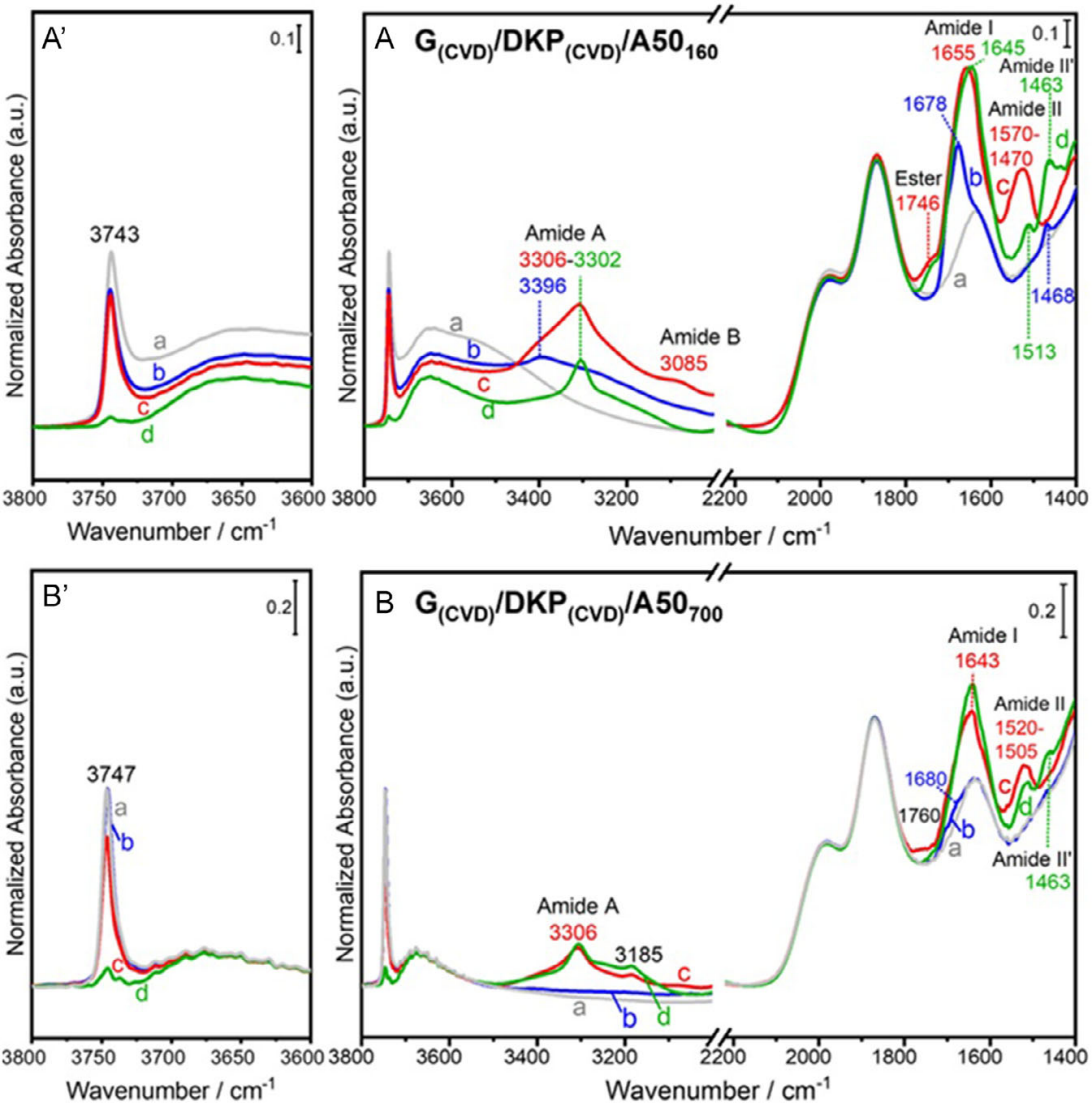
IR spectra measured on (A) G_(CVD)_/DKP_(CVD)_/A50_160_ and (B) G_(CVD)_/DKP_(CVD)_/A50_700_: (a) after outgassing at 160 °C for 2 h under vacuum (Panel A) or pretreatment in a muffle furnace at 700 °C for 2.5 h (Panel B), (b) after DKP deposition by CVD for 2.5 h, (c) after 10 (Panel A) or 5 h (Panel B) of Gly sublimation by CVD with steps of 2.5 h, (d) after cycles of H/D exchange and then outgassing of D_2_O vapor at bt under invariance of spectra. In panels A’ and B’, the horizontal (wavenumber) scale is expanded for the sake of clarity.

The IR profiles of G_(CVD)_/DKP_(CVD)_/A50_160_ and G_(CVD)_/DKP_(CVD)_/A50_700_ samples after DKP deposition by CVD, subsequent Gly deposition by CVD for 5 or 10 h, and H/D exchange cycles and outgas at rt are displayed in Figure 4A,B, respectively. The IR spectra of bare A50 silica after outgassing at 160 °C or heating at 700 °C are also shown.

After DKP deposition by CVD for 2.5 h on the A50 silica substrates (A50_160_ and A50_700_), different behaviors are seen on each of the samples obtained. At low frequency, for G_(CVD)_/DKP_(CVD)_/A50_160_, the corresponding IR profile (Figure 4A, curve b) shows the formation of the characteristic bands of adsorbed DKP (amide I, DKP ring stretching, and amide A). On the contrary, the aforementioned bands are not evident on G_(CVD)_/DKP_(CVD)_/A50_700_ where the IR spectrum recorded after DKP deposition by CVD (Figure 4B, curve b) shows almost a similar profile as the one of the corresponding bare silica A50_700_ (curve a). This indicates that the DKP was not successfully adsorbed on A50 silica surface preheated at 700 °C. Accordingly, in the ν_OH_ region, the silanol pattern is affected by the presence of DKP molecules on the surface of G_(CVD)_/DKP_(CVD)_/A50_160_ while no significant change is seen in the silanols pattern of G_(CVD)_/DKP_(CVD)_/A50_700_ sample.

For G_(CVD)_/DKP_(CVD)_/A50_160_, a significant decrease of the broad band centered at 3330 cm^−1^, associated to H‐bonded silanols (considered to be distant by less than ∼3 Å), is accompanied by a decrease in the profile peaking at 3743 cm^−1^ (Figure 4A, curve a and A’, curve b, respectively), where weakly interacting silanols, separated by 4–6 Å, known as NFS are found [19, 20, 25]. This suggests that DKP molecules are adsorbed on the silica surface through an interaction with both H‐bonded silanols and NFS. As previously discussed by Rimola et al. [25], the thermal treatment of the A50 silica surface at high temperature (700 °C) results in the condensation of the NFS and H‐bonded silanols and the formation of new isolated silanol groups (separated by more than 6 Å) and siloxane rings. The absence of the characteristic bands of DKP on the A50_700_ surface gives further confirmation that a silica surface depleted from these silanol sites (H‐bonded and NFS) makes it a nonsuitable platform for the adsorption and reaction of DKP.

Here, it is important to highlight that according to literature, the diameter of a DKP molecule could be estimated to roughly 5.14 Å based on its structural features [24]. And since NFS groups can be spaced by 4–6 Å, they may be in close proximity to interact with the DKP molecule through hydrogen bonding or other intermolecular forces leading to its adsorption on the silica surface. This indeed highlights a selectivity in the DKP adsorption on specific active sites on the silica surface.

After a subsequent Gly monomers deposition by CVD, the IR profile of G_(CVD)_/DKP_(CVD)_/A50_160_ recorded after 10 h Gly CVD at 160 °C (Figure 4A, curve c) shows the formation of relatively intense amide I, amide II, amide A, and amide B bands, along with the absence of the characteristic bands of DKP that are no more seen at this step. This implies that DKP molecules have been opened by the mean of Gly monomers deposited from gas phase on A50_160_ to form linear peptides strongly bonded to the surface through ester groups, detected through the formation of a significant band at around 1746 cm^−1^ [20]. In the ν_OH_ region, the silanol patterns of both H‐bonded and NFS are scarcely affected (Figure 4A,A’, curve c): the intensities of the broad band at 3330 cm^−1^ and the peak at 3743 cm^−1^, respectively, barely decrease after Gly monomers deposition by CVD. This gives further confirmation that H‐bonded and NFS groups are still altered by the adsorbed DKP molecules that opened into linear oligomers at this stage.

On the other hand, for G_(CVD)_/DKP_(CVD)_/A50_700_ sample (Figure 4B, curve c), the amide I, amide II, and amide A band are an indication of the formation of some Gly oligomers on the surface of A50_700_. However, for this sample, the band of ester groups is no more evident but instead a subtle one is formed at around 1760 cm^−1^ along with a newly formed one at 3185 cm^−1^ not observed before for G_(CVD)_/DKP_(CVD)_/A50_160_. The aforementioned bands are more likely to be associated to ν_C=O_ in the COOH moiety and ν_NH_ of Gly monomers, respectively [14, 33]. In the silanol groups region, only a decrease in the intensity of the peak associated to isolated silanols (3747 cm^−1^) is seen (Figure 4B’, curve c). This implies that the few oligomers formed on A50_700_ surface are weakly bonded to the surface and interacting with isolated silanols.

The relative amount of oligomers formed on both silica surfaces (A50_160_ and A50_700_) can be evaluated from the integrated area of the amide I band of the IR spectra recorded after each 2.5 h Gly CVD (Figure S5). For both G_(CVD)_/DKP_(CVD)_/A50_160_ and G_(CVD)_/DKP_(CVD)_/A50_700_, the temporal evolution of peptide bands can be roughly fitted with straight lines with nonzero intercepts (Figure S5). On G_(CVD)_/DKP_(CVD)_/A50_160_, peptides are significantly more abundant than on G_(CVD)_/DKP_(CVD)_/A50_700_ for the same time as Gly CVD. Thus, the silica surface A50_160_ represents an efficient platform for the formation of linear oligomers as observed experimentally and suggesting that it may arise from the presence of crucial elements (NFS and H‐bonded silanols) for the adsorption, reaction, and opening of DKP into abundant peptides.

When comparing these samples (G_(CVD)_/DKP_(CVD)_/A50_160_ and G_(CVD)_/DKP_(CVD)_/A50_700_) with samples prepared by in situ Gly CVD but not subjected to a predeposition of DKP by sublimation (G_(CVD)_/A50_160_ and G_(CVD)_/A50_700_), the relative amount of peptides formed is far more important in the first case (Figure S5). This implies that DKP represents a beneficial intermediate product instead of a dead‐end product for the formation of linear peptides on silica surface.

Both G_(CVD)_/DKP_(CVD)_/A50_160_ and G_(CVD)_/DKP_(CVD)_/A50_700_ were then subjected to cycles of D_2_O admission/outgas to investigate the different changes in the IR bands (Figure 4, curves d). For G_(CVD)_/DKP_(CVD)_/A50_160_, the amide I band that has a small NH in‐plane bending component shifts to a lower wavenumber (from 1655–1645 cm^−1^) upon deuteration [34] while its intensity and shape remain almost intact: a precise analysis is challenging due to the probable coexistence of both deuterated and nondeuterated NH. A more evident change is seen for the amide II band that is attributed to a combination of NH in‐plane bending and CN stretching: The band is partly, but not entirely consumed, while a new band associated to the amide II’ of deuterated peptide linkage is seen at 1463 cm^−1^ (the strong displacement of the amide II band is due to its significant NH‐bending component). This suggests that a part of the peptides formed on A50_160_ resists the D_2_O exchange. The original band of ester groups located at 1746 cm^−1^ is almost unaltered; this is a further proof of the assignment of this band and that the linear peptides formed remain anchored on the silica surface by ester bonds, resisting by that the hydration and H/D exchange [20, 21]. Furthermore, in the ν_NH_ region, it is clear that for G_(CVD)_/DKP_(CVD)_/A50_160_ sample, the amide A band is composed of two components: One narrow located at around 3306–3302 cm^−1^ and another broad one at around 3400 cm^−1^. After H/D exchange (Figure 4A, curve d), the component at 3400 cm^−1^ completely disappeared while the narrow one resists the exchange. This suggests, according to our assignments in our previous studies [20, 21], that the amide links in the peptide chains belong to two different categories: one susceptible to D_2_O exchange (the broad component at 3400 cm^−1^) while the other (at around 3302 cm^−1^) is inaccessible and/or stabilized by H‐bonding. This sharp band that remains in the region of amide A after H/D exchange is a characteristic behavior of well‐ordered structures on the surface.

On the other hand, the amide I band of G_(CVD)_/DKP_(CVD)_/A50_700_ (Figure 4B, curve d) increases in intensity and changes in shape but remains at 1643 cm^−1^. The amide II band also disappears partly but not completely, resulting in the formation of amide II’ band at 1463 cm^−1^. However, the band originally formed at 1760 cm^−1^ disappears almost completely; which gives a further proof that it cannot be assigned to ester groups but indeed to ν_C=O_ in the COOH moiety of Gly monomers which can be easily desorbed from the silica surface during the cycles of hydration and D_2_O exchange [14]. In the region of ν_NH_ of the oligomer chains, the shape an intensity of amide A for G_(CVD)_/DKP_(CVD)_/A50_700_ remains almost unaltered by the H/D exchange. This suggests that the amide links in the few oligomers formed are stabilized by H‐bonding making them resistant to D_2_O exchange; however, they are probably bonded to the silica surface through H‐bonding instead of ester groups as the case of G_(CVD)_/DKP_(CVD)_/A50_160_.

**SCHEME 2:**
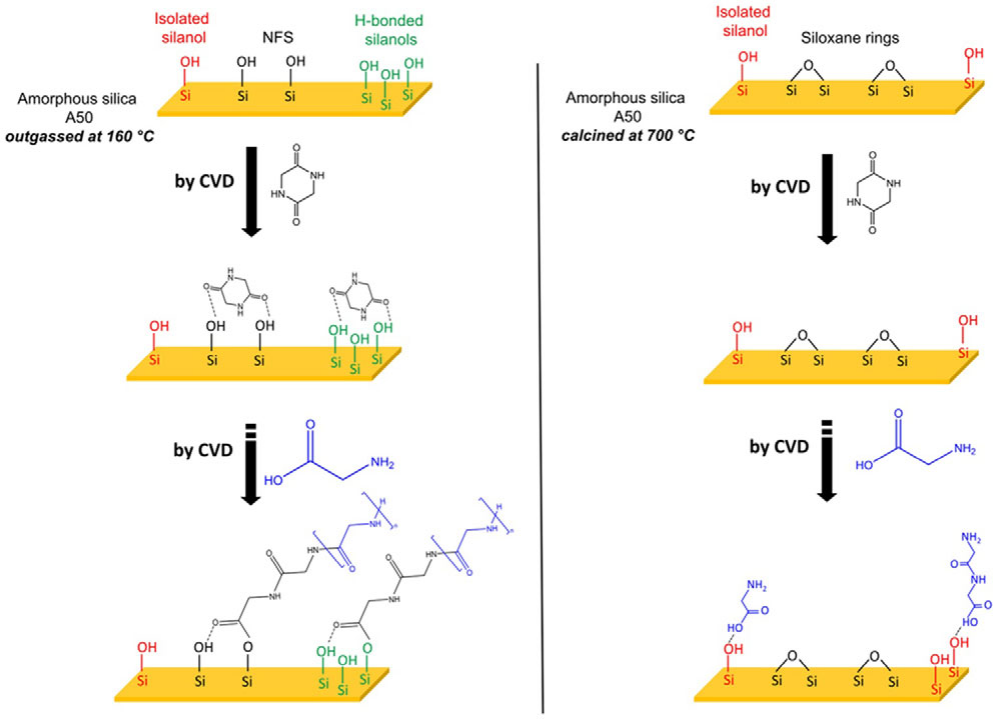
Schematic representation of the effect of different silanol groups on the opening of DKP and on the polymerization product obtained.

These behaviors on the two different silica surfaces in the frame of the polymerization reaction are sketchily summarized in Scheme 2.

After H/D exchange cycles, both samples G_(CVD)_/DKP_(CVD)_/A50_160_ and G_(CVD)_/DKP_(CVD)_/A50_700_ were subjected to XRD and TGA measurements (Figures S6 and S7). The XRD pattern of G_(CVD)_/DKP_(CVD)_/A50_160_ shows a comparable pattern to bare silica A50 (Figure S6A, curves b and a, respectively) which suggests that no crystalline Gly or peptides are present on the surface but instead only molecularly adsorbed species or chemically bonded ones without crystalline periodicity are formed. For G_(CVD)_/DKP_(CVD)_/A50_700_, the XRD pattern shows one peak that refers to bulk α‐Gly (2*θ* equals 18.7°). This was expected from the corresponding IR spectra (Figure 4B) that showed the presence of some crystalline Gly monomers on the surface: the majority might be removed by hydration and H/D cycles while a small amount is left and detected by XRD measurements.

DTG traces for both samples are also displayed in Figure S7 to discriminate what was really formed on the surface at the end of the H/D cycles. For G_(CVD)_/DKP_(CVD)_/A50_160_ (Figure S7A), one thermal event is observed at 321 °C and which can be associated to the oxidative degradation of the linear peptides anchored to the silica A50_160_; their amount can be estimated to 0.6% by weight through the integration of the corresponding band (Table S2).

For G_(CVD)_/DKP_(CVD)_/A50_700_ (Figure S7B), two different thermal events are observed. The first one occurring at around 235 °C and estimated to 2.2% by weight can be associated to the oxidative degradation of the shorter oligomers formed on A50_700_ and which are bonded to the surface through H‐bonding; in addition to some desorption of Gly monomers left on the surface. The second thermal event is observed at around 322 °C and corresponds to 1.1% by weight, which is almost twice that of the event on G_(CVD)_/DKP_(CVD)_/A50_160_. Although the event occurs at almost the same temperature as the first sample, however, it is impossible to attribute it to the thermal degradation of twice the amount of strongly bonded linear peptides, as proved by the IR spectra recorded (Figure 4). Consequently, it might be that, due to the temperature increase during the TGA measurement, some of the Gly monomers deposited on the surface of A50_700_ reacted to form DKP and thus this thermal event could be associated to the degradation of the newly DKP formed (as confirmed by the DTG trace of the pure DKP measured but not shown).

#### 2.2.2 CVD of DKP on Silica‐Grafted Gly Oligomers

**FIGURE 5:**
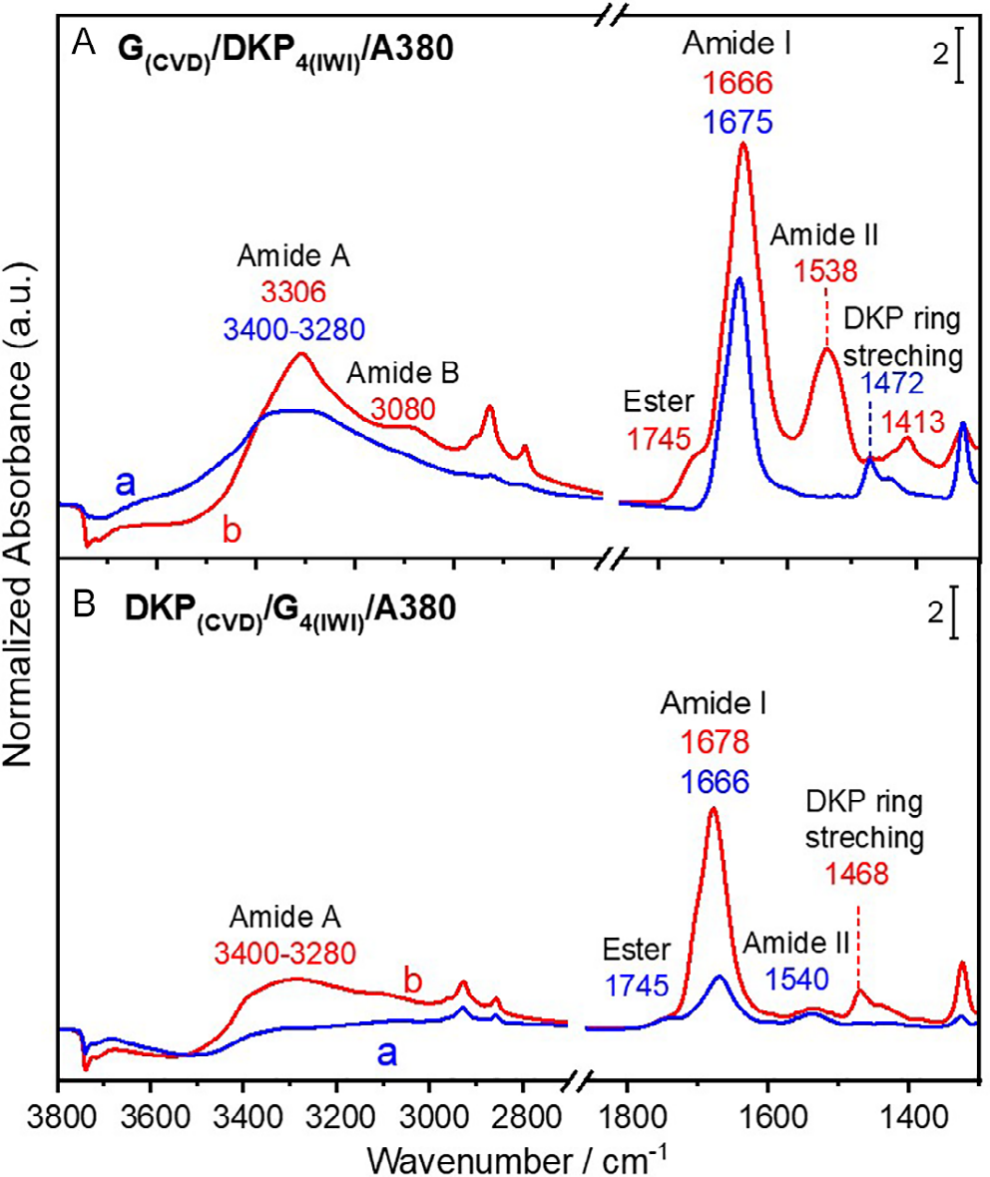
Panel (A) represents the IR difference spectra obtained (a) after 4 wt% DKP deposition by IWI and outgas at rt, and then (b) after Gly sublimation at 160 °C for 2.5 h under vacuum by CVD for G_(CVD)_/DKP_4(IWI)_/A380 sample. Same data as in Figure 1, panels (B) and (B’), curves (e) and (k), here reported for comparison. Panel (B) represents the IR difference spectra obtained (a) after 4 wt% Gly deposition by IWI and activation at 160 °C for 30 min, and then (b) after DKP sublimation at 160 °C for 2.5 h under vacuum by CVD for DKP_(CVD)_/G_4(IWI)_/A380 sample. The bare silica A380 subjected to distilled water impregnation by IWI and outgas at rt is subtracted as baseline for both panels.

To further assess the role of silica surface in the opening of the DKP ring, a new sample was prepared by depositing 4 wt% Gly on silica A380 using IWI procedure and followed by a thermal activation at 160 °C for 30 min under vacuum. Subsequently, DKP has been adsorbed on the sample from gas phase by CVD at 160 °C for 2.5 h. The resulting sample has been labeled as DKP_(CVD)_/G_4(IWI)_/A380. The difference IR spectra of DKP_(CVD)_/G_4(IWI)_/A380 recorded after each step are displayed in Figure 5 along with the ones of the sample G_(CVD)_/DKP_4(IWI)_/A380 previously discussed in relation to Figure 1.

For G_(CVD)_/DKP_4(IWI)_/A380, the difference IR spectrum recorded after DKP deposition by IWI on silica A380 (Figure 5A, curve a) have been discussed above in Figure 1B,B’. In brief, curve a (Figure 5A) recorded after IWI procedure shows the formation of the DKP characteristic bands (amide I, DKP ring stretching and amide A of the DKP ring [14, 18]. After Gly monomers adsorption by CVD (Figure 5A, curve b), the amide I band is shifted to a lower wavenumber (1666 cm^−1^) along with the appearance of amide II band (1538 cm^−1^). The amide A band becomes sharper and accompanied with amide B band (3080 cm^−1^). Along with the absence of DKP characteristic bands, this is an indication of the opening of the DKP rings to form linear oligomers, bonded to the surface through ester groups (1745 cm^−1^).

The DTG trace of G_(CVD)_/DKP_4(IWI)_/A380 sample (Figure S8A ) exhibits two peaks at 234 and 325 °C. In agreement with what discussed for the DTG and XRD results (Figures 2 and 3), these are related to the condensation of bulk Gly (2.45% by weight of silica, Table S3) and to the oxidative degradation of the linear peptides formed after DKP ring opening, respectively. In this case a significant amount of linear peptides is formed, quantified in 8.59% by weight.

On the other hand, for DKP_(CVD)_/G_4(IWI)_/A380, the difference IR spectrum recorded after 4 wt% Gly deposition on silica A380 by IWI followed by a thermal activation at 160 °C for 30 min (Figure 5B, curve a) shows the formation of both amide I (1666 cm^−1^) and amide II (1540 cm^−1^) bands along with the ester band around 1745 cm^−1^, indicating the formation of some Gly oligomers grafted to the surface by means of ester groups [18]. However, after DKP deposition from gas phase by CVD, the amide I band exhibits a significant shift to a higher wavenumber (1678 cm^−1^) while the amide II band (1540 cm^−1^) remains unaltered (Figure 5B, curve b). The absence of growth of the amide II band indicates the lack of the formation of new linear oligomers after DKP deposition from gas phase, especially when compared to G_(CVD)_/DKP_4(IWI)_/A380 (Figure 5A, curve b). In addition, the appearance of the band at 1468 cm^−1^ associated to the DKP ring stretching along with the formation of broad amide A band displayed in the range of 3400–3280 cm^−1^ (Figure 5B, curve b) give a further confirmation that the DKP deposited from gas phase by CVD does not open into linear oligomers, but instead simply adsorbs on the silica surface. The DTG trace of DKP_(CVD)_/G_4(IWI)_/A380 sample displayed in Figure S6B provides further evidence for this lack of reactivity. Two events at 275 and 322 °C account for adsorbed DKP [14, 18] (2.21%) and a small amount (2.41%) of strongly bonded Gly oligomers formed prior to DKP deposition by CVD (Table S3).

Here, it is important to underline that according to the XRD results in our previous study [18], the sample G_4(IWI)_/A380 was not saturated by Gly monomers before DKP deposition by CVD: The 4 wt% of Gly deposited by IWI represents only a fraction of the estimated saturation coverage of about 35 wt% on A380 silica. This excludes the fact that DKP deposited later by CVD on this surface does not open due to a saturated silica surface by Gly monomers but instead suggests the crucial role of some adsorption sites, already engaged in the interaction with Gly, for the DKP ring opening.

**SCHEME 3:**
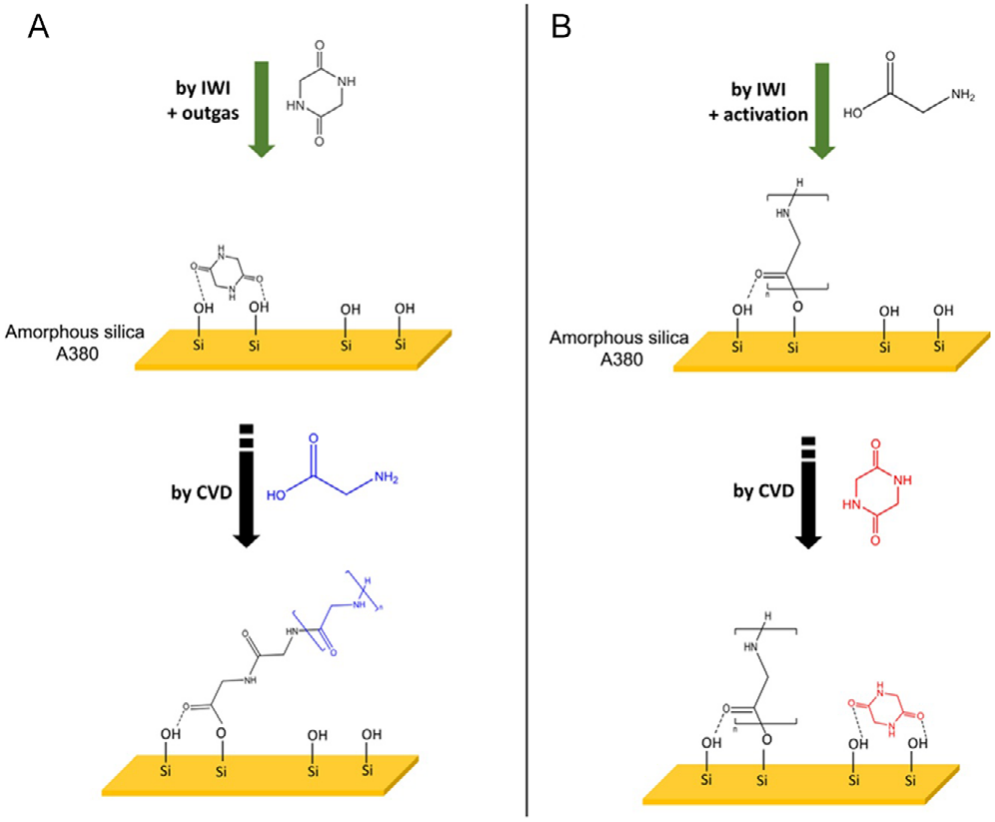
Schematic representation of the effect of the order of deposition of DKP and Gly on amorphous silica A380 on the product type of the polymerization reaction: (A) DKP firstly deposited by IWI on silica then Gly subsequently adsorbed from gas phase by CVD; in comparison with (B) Gly monomers firstly deposited by IWI on silica then DKP subsequently adsorbed by CVD. The interaction of DKP with surface silanols is only pictorial.

The two different scenarios observed on G_(CVD)_/DKP_4(IWI)_/A380 and DKP_(CVD)_/G_4(IWI)_/A380 samples are sketchily summarized in Scheme 3. The different types of products obtained when applying different DKP deposition procedures in this section strongly suggest that the silica surface and particularly some sort of silanol groups play an indispensable role in the opening of the DKP ring in the frame of the polymerization reaction, as discussed above.

#### 2.2.3 Structural Dynamics and Secondary Structures of Peptides Formed by DKP Opening on Silica

This section is dedicated to studying the structural dynamics (flexibility and degree of solvent accessibility) of the Gly oligomers formed after reaction of Gly monomers dosed from the gas phase with DKP adsorbed from IWI or CVD (Figures 1 and 4, respectively).

**FIGURE 6:**
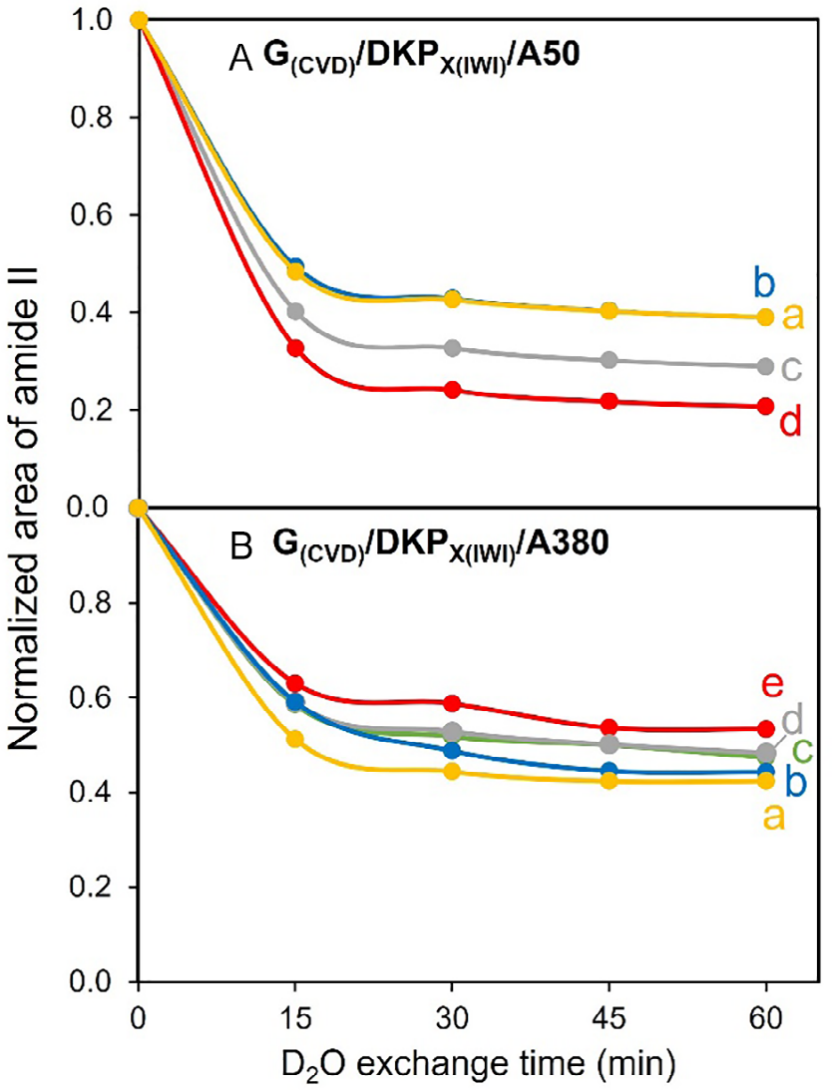
Evolution of the amide II band area during D_2_O adsorption/desorption (performed after 2.5 h Gly CVD) as function of D_2_O exchange time (min) for both series of samples (A) G_(CVD)_/DKP_*x*(IWI)_/A50 and (B) G_(CVD)_/DKP_*x*(IWI)_/A380, where (a) refers to the corresponding bare silica A50 or A380. The samples with different DKP loading are represented by (b) 1, (c) 2, (d) 3, (e) 4 wt%.

Both G_(CVD)_/DKP_*x*(IWI)_/A50 and G_(CVD)_/DKP_*x*(IWI_)/A380 (spectra reported in Figure 1) were subjected to water vapor contact followed by D_2_O adsorption/desorption cycles (graphs not shown). The kinetics of the H/D exchange in peptide links was followed by monitoring the residual intensity of the amide II band as function of the sample exposure to D_2_O during all the intermediate cycles of adsorption/desorption for a total of 60 min (Figure 6).

The amount of H/D exchange of amide II is higher in the series of samples G_(CVD)_/DKP_*x*(IWI)_/A50 than in G_(CVD)_/DKP_*x*(IWI)_/A380. For G_(CVD)_/DKP_*x*(IWI)_/A50 (Figure 6A), the amount of exchange of amide II becomes higher as the DKP weight loading increases: after the first 15 min of D_2_O adsorption/desorption cycles, only around 52% of the amide groups of the oligomers formed on bare silica (curve a) and on G_(CVD)_/DKP_1(IWI)_/A50 (curve b) were deuterated; while 70% were exchanged on G_(CVD)_/DKP_3(IWI)_/A50 (curve d).

On the contrary, on G_(CVD)_/DKP_*x*(IWI)_/A380 (Figure 6B), the amount of H/D exchange of amide II decreases with the increase of the DKP weight loading predeposited on the sample. For G_(CVD)_/A380, 49% of the amide groups were exchanged after the first 15 min of D_2_O admission/outgas cycles (curve a). This value decreases to 41% on G_(CVD)_/DKP_1(IWI)_/A380 (curve b) and becomes only 37% on G_(CVD)_/DKP_4(IWI)_/A380 (curve e).

The value at time 0 is recorded after the admission of water vapor for 20 min and outgas at rt; the other values are recorded after each 15 min of D_2_O admission and outgas until invariance of spectra.

As the kinetics of the amide H/D exchange could be related to the rigidity of the peptide secondary structures, the different types of the secondary structures that evolved after the 2.5 h Gly CVD and H/D cycles were quantified on both series of samples G_(CVD)_/DKP_*x*(IWI)_/A50 and G_(CVD)_/DKP_*x*(IWI)_/A380 (Figures S9 and S10, respectively) from the computation of the second derivative of the corresponding IR spectra. For G_(CVD)_/DKP_*x*(IWI)_/A50 series, β‐sheet (packed conformations) were formed at very low DKP weight loadings (*x* = 0 or 1 wt%) while only random coils disordered, shorter, and/or more flexible structures are detected on the sample with the high DKP weight loading (*x* = 3 wt%) (Figure S9A, Table S4). The H/D cycles applied (Figure S9B, Table S4) did not affect the β‐sheet structures on the samples with very low DKP weight loading but instead they become even more evident/aggregated. On G_(CVD)_/DKP_3(IWI)_/A50, some β‐turns (flexible structures) and β‐sheets start to form in addition to the presence of random coils after D_2_O admission/outgas [21, 35].

On the other hand, for G_(CVD)_/DKP_*x*(IWI)_/A380 series, only β‐turns were formed after 2.5 h Gly CVD on samples with low DKP weight loadings (*x* = 0 or 1 wt%) while β‐sheets in addition to some β‐turns were formed on the sample with higher loading (*x* = 4 wt%) (Figure S10A, Table S4). After the H/D exchange, random coils start to be observed on the sample with no DKP (*x* = 0 wt%). On the other hand, β‐sheets are formed with increasing DKP weight loadings after H/D cycles applied, highlighting by that that the presence of DKP as an intermediate product during polymerization reaction results in the formation of more abundant and longer chains that can be packed into β‐sheets structures upon contact with water vapor (Figure S10B, Table S4) [21, 35].

## 3 Conclusions

In comparison with the limited number of previous studies dealing with the cyclic anhydride DKP, the novelty of the present work lies first in an in‐depth study of different conditions for DKP opening in the frame of the Gly polymerization on silica surfaces of low and high surface areas (A50 and A380, respectively). DKP, deposited on both silica surfaces from liquid phase by IWI followed by Gly monomers adsorption from gas phase by CVD, plays an efficient role as an intermediate for the formation of peptides. These linear peptide chains are formed in higher abundance when compared with the case of DKP absence on silica surface and show a highly organized secondary structures including mainly β‐sheet that resist deuterium exchange. On high‐surface‐area silica, the abundancy of the resulting linear peptides increases with the increase of DKP loading (from 1 to 4 wt%). On the contrary, due to surface coverage on low‐surface‐area silica, more linear peptides are formed when low DKP loading (1 wt%) is used.

On the other hand, DKP, deposited from gas phase by CVD on silica surface pregrafted with linear oligomers, does not open to form additional oligomeric chains but instead simply adsorbs on the surface; this highlights that the DKP interaction with surface silanols is crucial for ring opening to promote further peptide formation.

Conditions of wetting/drying cycles in controlled vacuum are also tested for DKP opening on silica. Surprisingly, DKP, adsorbed on the silica surface from gas or liquid phase, shows a high resistance to hydrolysis contrary to what was mentioned in literature when working instead in water–silica solutions.

In CVD conditions, under controlled atmosphere, DKP (of approximately 5.14 Å as diameter) seems to show a high selectivity toward nearly free silanol (NFS) groups distant by 4–6 Å. This special type of silanols with which the DKP ring interacts through hydrogen bonding or other intermolecular forces, seems to play a crucial role in DKP adsorption, reactivity, and opening into linear peptide chains on the silica surface.

These results show that the cyclic anhydride DKP is far from an uninteresting or dead‐end product; instead, it appears to be beneficial for peptide formation on silica surfaces. In this work, the different experimental conditions for DKP opening and the crucial role of NFS silanols on silica for its adsorption, and reaction to form linear oligomers are highlighted for the first time based on a combination of IR spectroscopy,TGA, and X‐ray diffraction (XRD). Solid‐state NMR could also be an interesting technique for future studies dealing with the elucidation of DKP surface chemistry on silica and bridging it to the study of such compounds in the origin of life scenarios.

## 4 Experimental Section

### 4.1 Materials

The commercial highly pure pyrogenic silica powders Aerosil OX 50 and Aerosil A380 (designated as A50 and A380) of nominal specific surface areas 50 and 380 m^2^.g^−1^, respectively, provided by Evonik, SiO_2_ content ≥99.8 wt%), were used as supports in the present work. Natural abundance glycine and glycine anhydride (2,5‐diketopiperazine, DKP) (99%), provided by Sigma–Aldrich were used as received. Deuterated water D_2_O (99.90 atom % D), a high‐purity product purchased from Sigma–Aldrich, and Milli‐Q water (Millipore system) were admitted in the IR cell through the vacuum line after several freeze–pump–thaw cycles.

### 4.2 Methods

#### 4.2.1 DKP or Gly Adsorption on Silica Supports From the Liquid Phase

DKP units (or Gly monomers for Part A.2) were deposited on silica surfaces from water solutions using the procedure of the IWI, derived from the field of the synthesis of supported catalysts. In short, the required amount of DKP units was dissolved in ultrapure water, and the obtained DKP solution was added to the silica support respecting a ratio of 10 ml of DKP solution for 1 g of silica [18]. The resulting homogeneous slurry obtained without a separate liquid phase was left for drying overnight at room temperature (rt) under a gentle flow of compressed air.

For each type of silica surface, a series of samples with increasing DKP weight loadings from 1 to 3 or 4% was prepared. For the reference samples prepared without DKP, a corresponding volume of ultrapure water was added to the bare silica A50 or A380. The DKP/silica systems (or the reference samples of A50 and A380) were pressed in the form of self‐supporting pellets where each one was put in a gold frame as a holder and introduced in a conventional IR cell. This cell was equipped with a valve to connect it to vacuum lines (residual pressure 1 × 10–5 mbar) and composed of two main parts: one dedicated to thermal treatment and the other was an IR‐transparent part with CaF_2_ windows for in situ IR spectroscopic measurements in transmission mode. The temperature during the thermal treatment was measured by means of a thermocouple placed in contact with the external surface of the cell. All DKP/silica samples were outgassed (dehydrated) under vacuum at rt for 2 h in the IR cell. Subsequently, only the DKP/silica A50 systems were further subjected to an outgas at 140 °C for 30 min under vacuum. These samples were referred to as DKP_*x*(IWI)_/A_*y*_, where *x* represents the DKP (or Gly) weight loading and *y* refers to the specific surface area of the pristine silica used.

#### 4.2.2 Gly or DKP Adsorption on Silica Supports From the Gas Phase

Gly (or DKP in Parts A.2, A.3, and B) sublimation and adsorption on silica surfaces on DKP/silica systems was performed in situ in the IR cell using CVD [20, 21, 22]. Briefly, after outgassing at rt under vacuum, the sample (A50, A380, DKP_*x*_/A50, DKP_*x*_/A380, or G_4(IWI)_/A380) was moved to the thermal treatment part of the IR cell where it was heated up to 160 °C for 2.5 h in static vacuum next to a Gly (or DKP, according to the experiment) pellet of 200 mg which started to sublimate and adsorb on the substrate pellet. To get rid of the water vapor formed during Gly (or DKP) condensation reaction, a cold trap filled with liquid nitrogen was kept in contact with the cell. The valve connecting the cell to the vacuum line was closed to ensure that the Gly vapor remained inside the cell. After 2.5 h, the temperature was decreased to rt, and the sample pellet was moved to the transparent part for IR measurement. In part B of the present study, the sequence (contact with Gly vapor then IR spectra measurement) was repeated until reaching 10 h of sublimation in total (steps of 2.5 h).

The sublimation procedure by CVD was followed by (1) contact with H2O vapor for 15 min followed by outgas for 15 min at beam temperature (bt) (ca. 50 °C), and (2) H/D exchange through D_2_O vapor exposure for 15 min followed by outgas for 15 min at bt. The D_2_O adsorption/desorption cycle was repeated until invariance of the IR spectra recorded.

In part A.3 of the work, the samples were further subjected to wetting/drying (W/D) cycles that consisted of H_2_O vapor exposure for 15 min followed by a heating under vacuum at 80 °C for 30 min while in contact with H_2_O vapor. Subsequently, the samples were dried under vacuum at rt then dried while heating at 80 °C for 30 min. The W/D cycles are repeated until the invariance of IR spectra is recorded.

#### 4.2.3 Thermal Treatment of Silica

In part B of the work, the silica samples were subjected to a pretreatment prior to the sublimation procedure. Silica AOX50 powder was pressed in the form of two self‐supporting pellets denoted as A50_160_ and A50_700_. The first sample A50_160_ was put in a gold frame as a holder and inserted in the IR cell connected to a conventional vacuum line where it was just outgassed at 160 °C for 2 h to attain a complete surface dehydration before the start of the DKP and Gly adsorption and polymerization reaction.

The second A50_700_ pellet was introduced in a muffle furnace for thermal treatment at 700 °C according to the following conditions: The temperature was increased from rt to 450 °C with a ramp of 30 min and was kept at this temperature for 2.5 h. The temperature was then ramped for 30 min from450 to 700 °C and kept at this temperature for 2.5 h, then left in the furnace to cool down to rt before starting with the DKP and Gly sublimation reactions.

### 4.3 Characterization Techniques

#### 4.3.1 IR Spectroscopy

Throughout the different procedures, the various samples were monitored by means of in situ IR spectroscopy. IR spectra were recorded using a Bruker Vector 22 spectrometer equipped with a mercury cadmium telluride (MCT) detector, setting a resolution of 4 cm^−1^, and accumulating 128 scans to obtain a good signal to noise ratio.

#### 4.3.2 XRD

The samples were characterized by X‐ray powder diffraction patterns recorded on a PANalytical X'pert PRO powder diffractometer featuring Bragg–Brentano geometry and using a Cu Kα (*λ* = 1.5405 Å) radiation source. Data were recorded for 2*θ* angles ranging from 10° to 40° with a step size of 0.01°and a dwell time of 120 s per step.

#### 4.3.3 TGA

Thermograms of the different samples were collected on the corresponding crushed pellets using a TA instrument paired with a STD Q600 analyzer. The ground samples were heated up to 600 °C following a heating ramp of 10 °C.min^−1^ under a dry air flow of 100 ml.min^−1^. Quantification of adsorbed products (DKP or peptides) was evaluated by correcting the weight loss between 120 and 600 °C for the corresponding values of the blank sample.

## Conflicts of Interest

The authors declare no conflicts of interest.

## Supporting information

Supplementary Material

## Data Availability

The data that support the findings of this study are available from the corresponding author upon reasonable request.
