# Supplementary material for: The Role of Cyclic Diketopiperazine in the Formation of Polypeptides on Silica Surfaces
Source: Chempluschem. 2026 Jul 26;91(7):e70216. doi: 10.1002/cplu.70216 (PMC13402219; doi:10.1002/cplu.70216)
Supplement: Supplementary file 1 — Supplementary Material [file CPLU-91-e70216-s001.pdf]

Supporting information

The role of cyclic diketopiperazine in the formation of polypeptides on silica surfaces

Ola El Samrout*, Rita Arnesi, Samuele Mistrali, Chiara Nannuzzi, Gloria Berlier*.

Department of Chemistry, NIS and INSTM Centers, University of Turin, via P. Giuria 7, 10125 Turin, Italy

DKP deposited on silica from liquid phase by IWI

Thermal activation

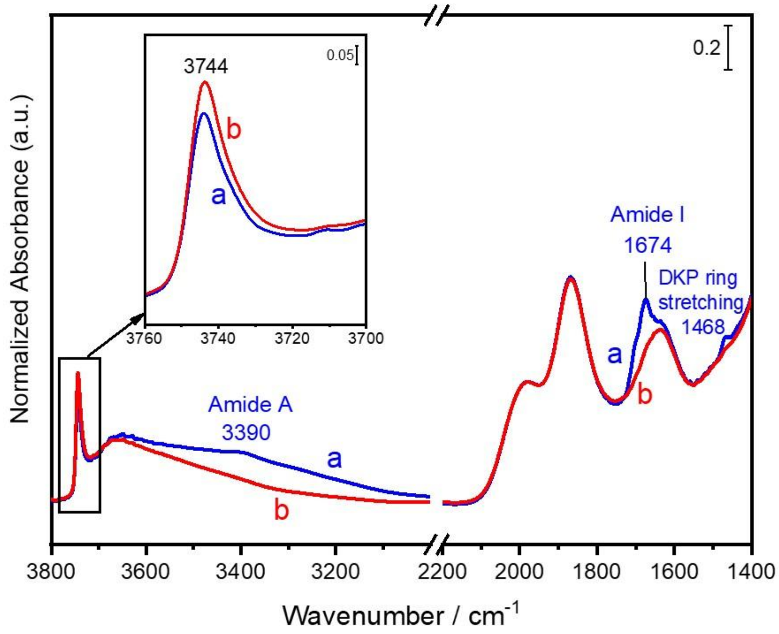

Figure S1. IR spectra of $DKP_{2(IWI)}$/A50 measured after (a) DKP deposition by IWI followed by outgas at 140 °C for 30 min, then after (b) a thermal heating at 160 °C for 2.5 h under vacuum. The intensity of the silanols OH stretching region is enhanced for the sake of clarity.

Wetting/drying cycles

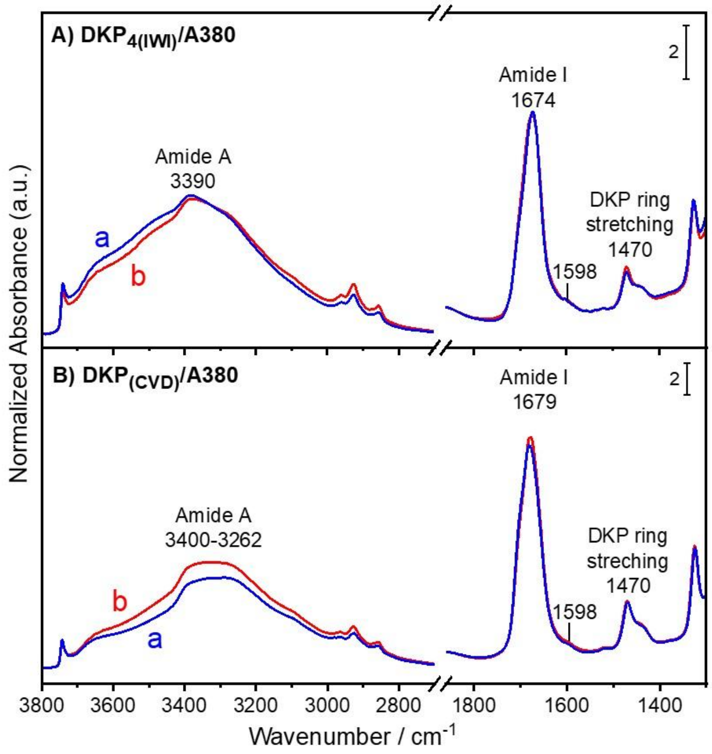

Figure S2. IR spectra of $DKP_{4(IWI)}$/A380 (Panel A) and $DKP_{(CVD)}$/A380 (Panel B) measured after (a) DKP deposition by IWI and outgas at rt, or after DKP sublimation by CVD for 2.5 h at 160 °C under vacuum, respectively. Spectra (b) in both panels represent each the last spectrum measured at the end of the wetting/drying cycles performed on both samples. The wetting/drying cycles done consist of an admission of water vapor for 20 min and a direct heating at 80 °C for 30 min while in contact with water vapor. This is followed by a drying step consisting of an outgas at rt then at 80 °C for 30 min. The cycles are repeated until invariance of spectra.

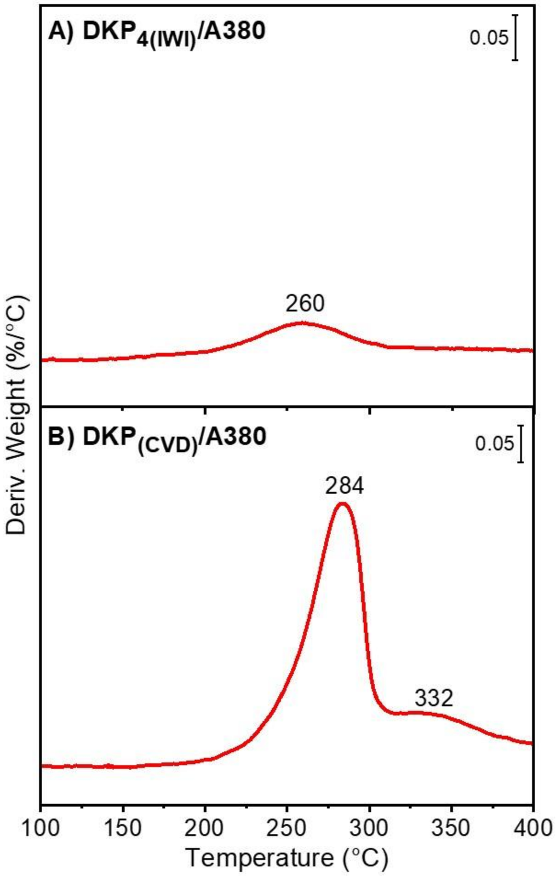

Figure S3. Derivative thermograms (DTG) measured directly after wetting/drying cycles performed on two samples prepared by (A) 4 wt% DKP deposition by IWI and outgas on silica A380, designated as $DKP_{4(IWI)}$/A380; or by (B) DKP sublimation by CVD for 2.5 h at 160 °C under vacuum, designated as DKP $_{(CVD)}$/A380.

Sample	Peak temperature (°C)	Adsorbed organic matter	
		Weight loss (%)	mmol DKP/g SiO_2
$DKP_{4(IWI)}$/A380	260	2.96	0.26
$DKP_{(CVD)}$/A380	284	22.52	1.97
	332	5.72	0.50

Table S1. Table presenting the adsorbed amount of organic matter (in terms of DKP) after wetting/drying cycles on both samples $DKP_{4(IWI)}$/A380 obtained after DKP deposition by IWI on silica A380; and DKP $_{(CVD)}$/A380 obtained after DKP sublimation by CVD for 2.5 h under vacuum. These values are calculated from the integration of DTG peaks of Figure S3 for the corresponding samples.

Reaction with Gly monomers dosed from the gas phase

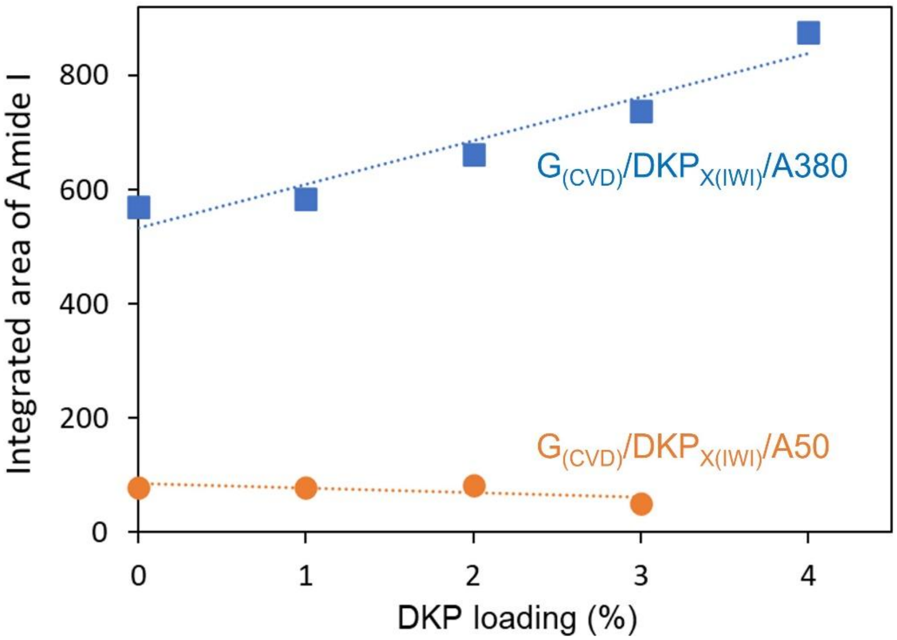

Figure S4: Evolution of the amide I band intensity obtained after 2.5 h Gly sublimation by CVD as function of DKP loading (%) for both series of samples: $G_{(CVD)}/DKP_{x(IWI)}/A50$ and $G_{(CVD)}/DKP_{x(IWI)}/A380$; where x refers to the different DKP loadings used.

DKP deposited on silica from gas phase by CVD

Reaction with Gly monomers dosed from the gas phase

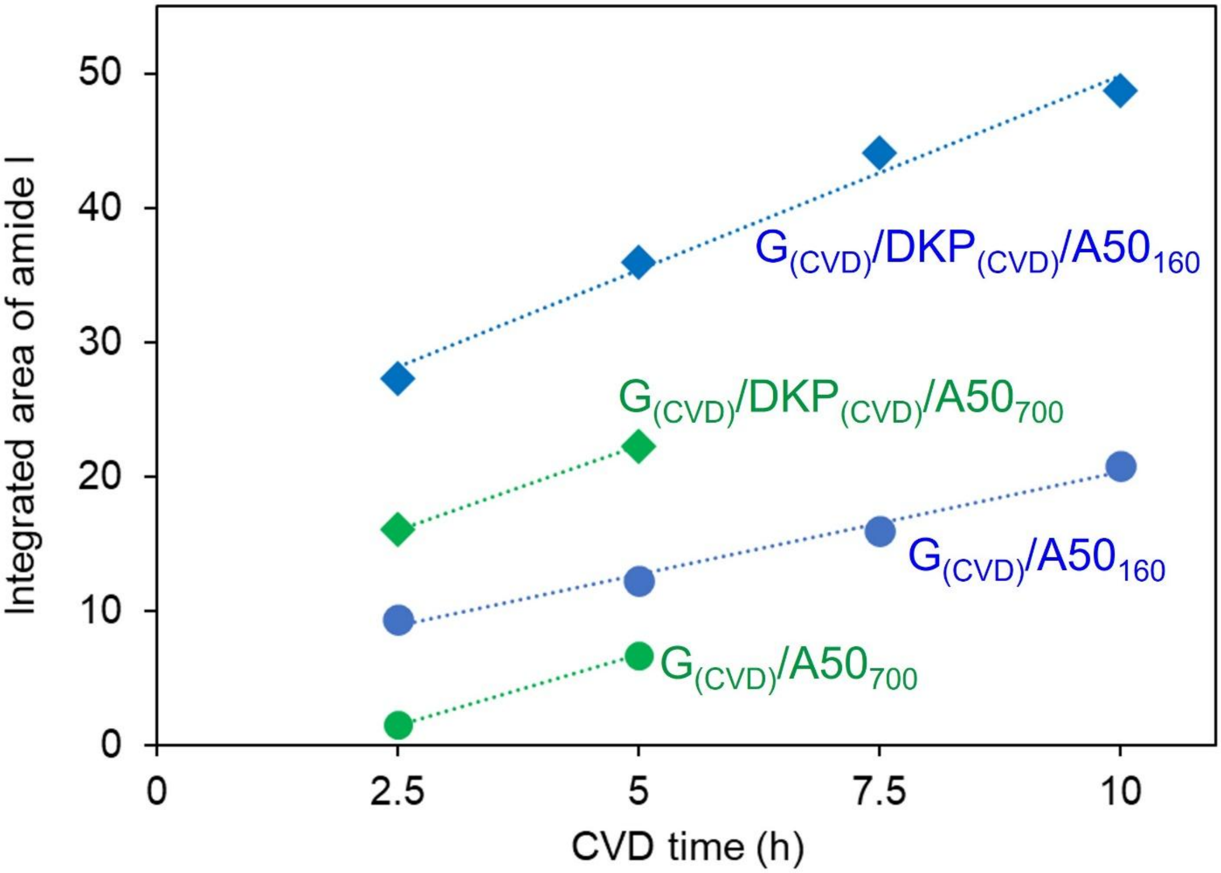

Figure S5: Evolution of the amide I band intensity as function of CVD time (h) for $G_{(CVD)}/A50_{160}$, $G_{(CVD)}/DKP_{(CVD)}/A50_{160}$, $G_{(CVD)}/A50_{700}$, and $G_{(CVD)}/DKP_{(CVD)}/A50_{700}$ samples prepared by in-situ CVD with or without a pre-deposition of DKP by sublimation. Two different silica supports are used: one outgassed at 160 °C for 2 h under vacuum and the other pre-treated at 700 °C for 2.5 h.

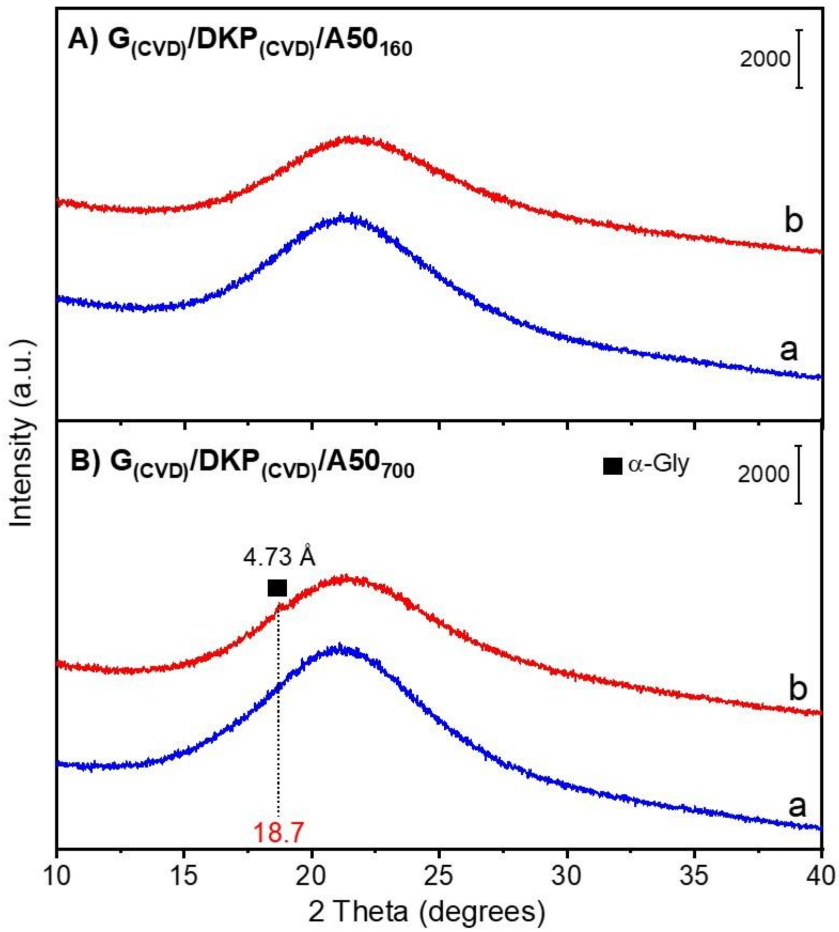

Figure S6: XRD profiles measured on (A) $G_{(CVD)}/DKP_{(CVD)}/A50_{160}$ and (B) $G_{(CVD)}/DKP_{(CVD)}/A50_{700}$: (a) after outgassing at 160 °C for 2 h under vacuum (Panel A) or pre-treatment in a muffle furnace at 700 °C for 2.5 h (Panel B); (b) after DKP deposition by CVD for 2.5 h under vacuum followed by Gly sublimation by CVD for 10 (Panel A) or 5 h (Panel B) and H/D exchange cycles.

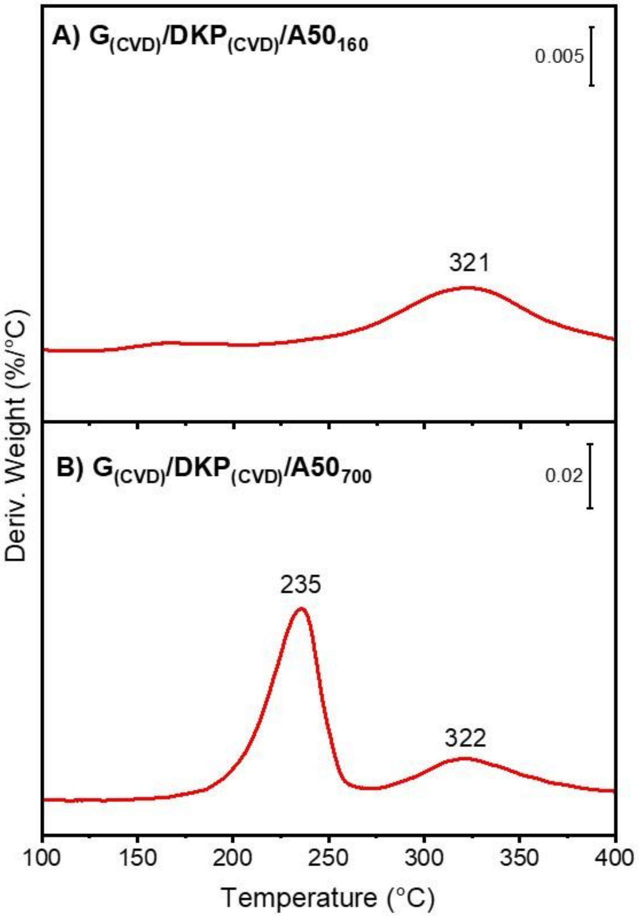

Figure S7: Derivative thermograms (DTG) for (A) $G_{(CVD)}/DKP_{(CVD)}/A50_{160}$ and (B) $G_{(CVD)}/DKP_{(CVD)}/A50_{700}$ samples measured after DKP deposition by CVD for 2.5 h under vacuum followed by Gly sublimation by CVD for 10 (Panel A) or 5 h (Panel B) and H/D exchange cycles.

Sample	Peak temperature (°C)	Adsorbed organic matter		
		Weight loss (%)	mmol g Gly/g SiO_2	mmol DKP/g SiO_2
$G/DKP/A50_{160}$	321	0.60	0.08	-
$G/DKP/A50_{700}$	235	2.23	0.29	-
	322	1.10	-	0.09

Table S2: Table presenting the adsorbed amount of organic matter (in terms of DKP or Gly) on both $G_{(CVD)}/DKP_{(CVD)}/A50_{160}$ and $G_{(CVD)}/DKP_{(CVD)}/A50_{700}$ samples. These values are calculated from the integration of DTG peaks of Figure S7 for the corresponding samples.

CVD of DKP on silica-grafted Gly oligomers

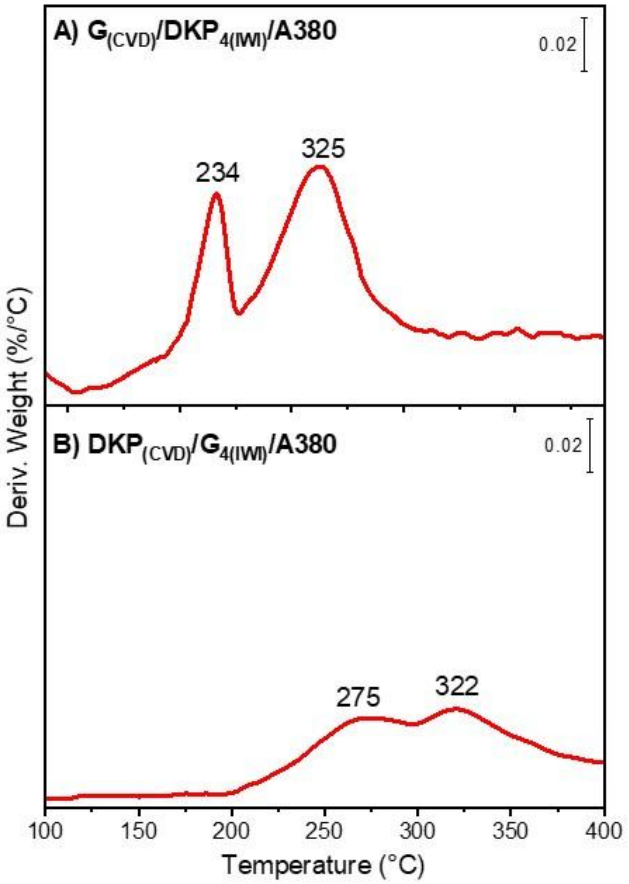

Figure S8. Derivative thermograms (DTG) for samples obtained after (A) 4 wt% DKP deposition by IWI and outgas on silica A380 followed by CVD sublimation of Gly monomers at 160 °C for 2.5 h under vacuum, designated by $G_{(CVD)}$/$DKP_{4(IWI)}$/A380; and after (B) 4 wt% Gly deposition by IWI and activation at 160 °C on silica A380 followed by DKP adsorption by CVD at 160 °C for 2.5 h under vacuum, designated by $DKP_{(CVD)}$/$G_{4(IWI)}$/A380.

Sample	Peak temperature (°C)	Adsorbed organic matter		
		Weight loss (%)	mmol g Gly/g SiO_2	mmol DKP/g SiO_2
$G_{(CVD)}/DKP_{4(IWI)}/A380$	234	2.45	0.33	-
	325	8.59	1.14	-
$DKP_{(CVD)}/G_{4(IWI)}/A380$	275	2.21	-	0.19
	322	2.41	0.32	-

Table S3. Table presenting the adsorbed amount of organic matter (in terms of DKP or Gly) on both samples $G_{(CVD)}/DKP_{4(IWI)}/A380$ obtained after 4 wt% DKP deposition by IWI and outgas then Gly sublimation by CVD for 2.5 h; and $DKP_{(CVD)}/G_{4(IWI)}/A380$ obtained after 4 wt% Gly deposition by IWI and activation at 160 °C then DKP sublimation by CVD for 2.5 h at 160 °C under vacuum. These values are calculated from the integration of DTG peaks of Figure S6 for the corresponding samples.

Structural dynamics and secondary structures of peptides formed by DKP opening on silica

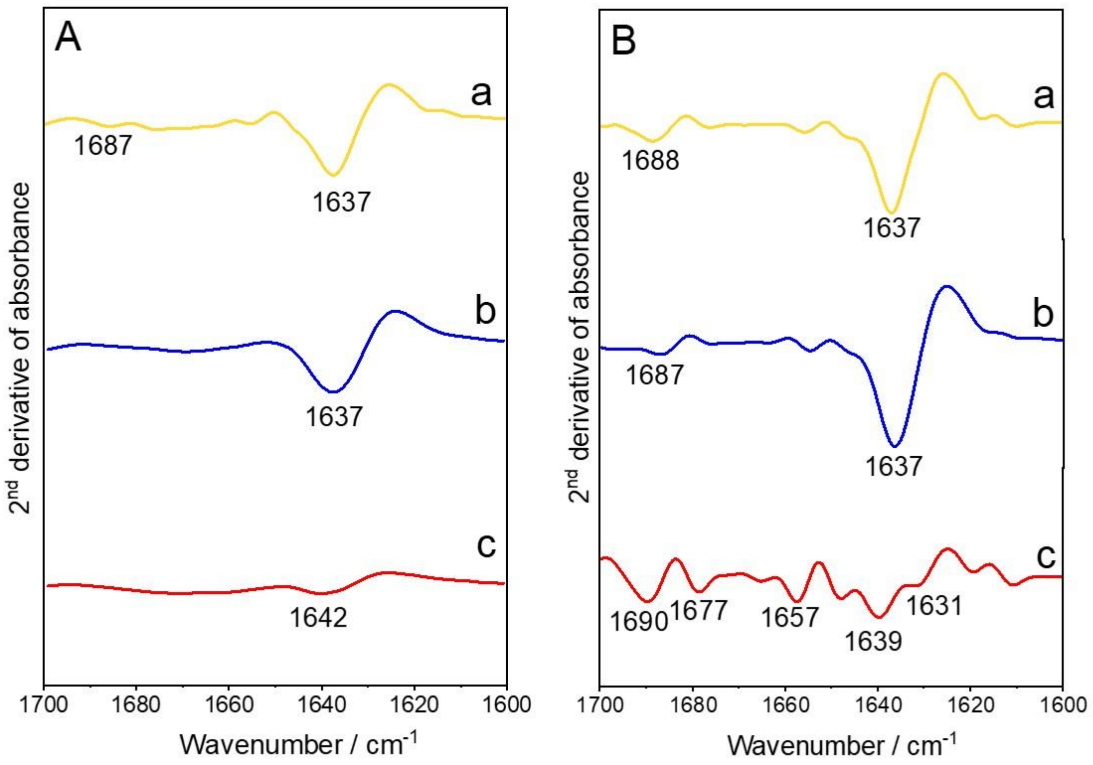

Figure S9. Second derivative in the amide I region of the IR spectra recorded directly after (Panel A) 2.5 h of Gly sublimation by CVD, and then after (Panel B) H/D exchange and outgassing of D_2O until invariance of spectra of (a) bare silica A50, (b) $G_{(CVD)}$/$DKP_{1(IWI)}$/A50, and (c) $G_{(CVD)}$/$DKP_{3(IWI)}$/A50.

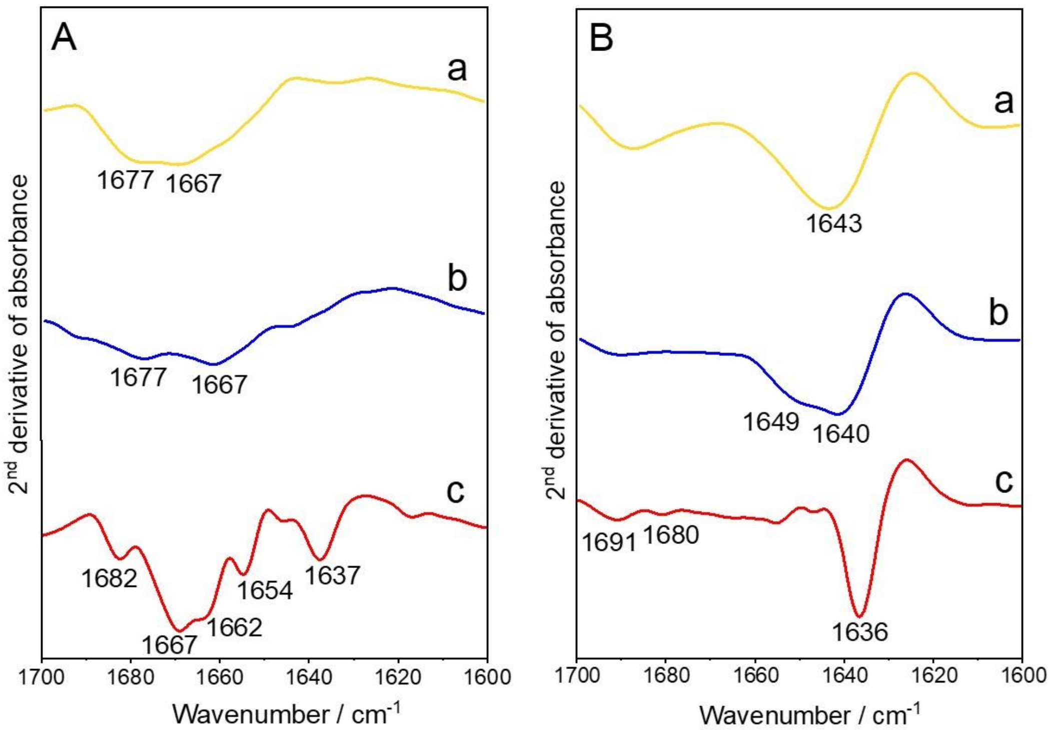

Figure S10. Second derivative in the amide I region of the IR spectra recorded directly after (Panel A) 2.5 h of Gly sublimation by CVD, and then after (Panel B) H/D exchange and outgassing of D_2O until invariance of spectra of (a) bare silica A380, (b) $G_{(CVD)}$/$DKP_{1(IWI)}$/A380, and (c) $G_{(CVD)}$/$DKP_{4(IWI)}$/A380.

Sample	wt% DKP	Secondary structures			
		**After 2.5 h CVD**		**After H/D cycles**	
		Assignment	Wavenumber (cm^{-1})	Assignment	Wavenumber (cm^{-1})
$G_{(CVD)}$/$DKP_{x(IWI)}$/A50	0	β- sheet	1637, 1687	β- sheet	
	1	β- sheet		more β- sheet	1637, 1687
	3	random coil	1642	β- sheet	1690;
				+ β- turn	1677;
				random coil	1639, 1631
$G_{(CVD)}$/$DKP_{x(IWI)}$/A380	0	β- turn	1677, 1667	random coil	1643;
	1	β- turn		β- sheet	
	4	β- sheet	1682, 1637;	β- sheet	1691, 1680, 1649,
		+ β- turn	1667, 1662		1637

Table S4. Table presenting the different types of secondary structures formed after 2.5 h CVD then after H/D cycles for both sets of samples $G_{(CVD)}$/$DKP_{x(IWI)}$/A50 and $G_{(CVD)}$/$DKP_{x(IWI)}$/A380, deduced from the computation of the second derivative of the corresponding IR spectra in Figures S7 and S8.
